# The Clinical Significance and Potential Molecular Mechanism of Upregulated CDC28 Protein Kinase Regulatory Subunit 1B in Osteosarcoma

**DOI:** 10.1155/2021/7228584

**Published:** 2021-12-10

**Authors:** Chaohua Mo, Le Xie, Chang Chen, Jie Ma, Yingxin Huang, Yanxing Wu, Yuanyuan Xu, Huizhi Peng, Zengwei Chen, Rongjun Mao

**Affiliations:** ^1^Department of Pathology, Foshan Hospital of Traditional Chinese Medicine, Guangzhou University of Chinese Medicine, Foshan, Guangdong 528300, China; ^2^Department of Pathology, Wuzhou Res Cross Hospital, Wuzhou, Guangxi Zhuang Autonomous Region 543100, China; ^3^Department of Medical Oncology, The First Affiliated Hospital of Guangxi Medical University, Nanning, Guangxi Zhuang Autonomous Region, China

## Abstract

**Background:**

CDC28 Protein Kinase Regulatory Subunit 1B (CKS1B) is a member of cyclin-dependent kinase subfamily and the relationship between CKS1B and osteosarcoma (OS) remains to be explored.

**Methods:**

80 OS and 41 nontumor tissue samples were arranged to conduct immunohistochemistry (IHC) to evaluate CKS1B expression between OS and nontumor samples. The standard mean deviation (SMD) was calculated based on in-house IHC and tissue microarrays and exterior high-throughput datasets for further verification of CKS1B expression in OS. The effect of CKS1B expression on clinicopathological and overall survival of OS patients was measured through public high-throughput datasets, and analysis of immune infiltration and single-cell RNA-seq was applied to ascertain molecular mechanism of CKS1B in OS.

**Results:**

A total of 197 OS samples and 83 nontumor samples (including tissue and cell line) were obtained from in-house IHC, microarrays, and exterior high-throughput datasets. The analysis of integrated expression status demonstrated upregulation of CKS1B in OS (SMD = 1.38, 95% CI [0.52–2.25]) and the significant power of CKS1B expression in distinguishing OS samples from nontumor samples (Area under the Curve (AUC) = 0.89, 95% CI [0.86–0.91]). Clinicopathological and prognosis analysis indicated no remarkable significance but inference of immune infiltration and single-cell RNA-seq prompted that OS patients with overexpressed CKS1B were more likely to suffer OS metastasis while MYC Protooncogene may be the upstream regulon of CKS1B in proliferating osteoblastic OS cells.

**Conclusions:**

In this study, sufficient evidence was provided for upregulation of CKS1B in OS. The advanced effect of CKS1B on OS progression indicates a foreground of CKS1B as a biomarker for OS.

## 1. Introduction

Osteosarcoma (OS) is a kind of malignant tumor originates from bone, accounting for 56% of the primary bone tumors, with an incidence rate of 3/10^5^ [1–4]. The frequency of OS showed a bimodal distribution pattern: the first peak appears at the age of 10∼14, and then the second arises after 60 [5–7]. Due to the research progress in the following three aspects, the overall cure rate of OS has been effectively improved in the past few decades: ① pathogenesis from the perspective of molecular pathway [8, 9]: recent studies have shown that inhibiting the activity of MYC Protooncogene (MYC) can reduce the proliferation and infiltration of OS cells and improve drug sensitivity [10, 11]. What is more, insulin growth factor (IGF) signaling pathway has been proved to be a vital part of the OS pathogenesis [12, 13]. IGF-1 receptor (IGF-1R) is a member of tyrosine kinase family while, after being activated by ligand IGF-1, it can promote cell proliferation, protein synthesis, and glucose metabolism and maintain tissue homeostasis and growth in OS [14, 15]. ② Key factors involved in OS metastasis: tumor cells migrating away from the primary lesion and invading into the blood vessel through extracellular matrix (ECM) are decisively for tumor metastasis. A few of studies have confirmed that matrix metalloproteinases 2 and 9 (MMP-2, MMP-9) and calcium-activated neutral proteinase 2 (CAPN2) play an important role in the degradation of extracellular matrix (ECM) in OS [16, 17]. ③ Exploration of potential therapeutic targets: epidermal growth factor receptor (EGFR) blocker, specific cell marker monoclonal antibody, and the antitumor angiogenesis drugs have proved their efficacy and made progress in early clinical trials of OS [18–22].

However, the current research on OS is still not yet thorough while a few dilemmas remain to be explored.① OS patients suffering deferred or inexact diagnosis endured a higher risk of losing chance for receiving standard treatment [23, 24], so effective molecular biomarker is especially needed.② A more delicate understanding of the cytological composition of OS is required to assist the OS patients with unidentified histological type [25] in acquiring specific intervention. ③ The mechanism of OS metastasis is not distinct [26–29] while the medical treatment for OS patients with lung metastasis presented poor curative effect [30], which demands penetrating knowledge of OS metastasis.

To promote and enrich researches on the above dilemma, focus point of this study is concentrated on CDC28 Protein Kinase Regulatory Subunit 1B (CKS1B) through preliminary work containing literature review and data screening. CKS1B mRNA serves as a necessary part of S-Phase Kinase Associated Protein 2 (Skp2) ubiquitination complex while Skp2 specifically recognizes phosphorylated substrates and mediates their ubiquitination degradation; however, many cell cycle regulators are substrates of ubiquitin proteasome pathway [31, 32]. CKS1B promotes the binding of Skp2 to phosphorylated cyclin-dependent kinase inhibitor 1B (CDKN1B), one of the main target molecules of Skp2, resulting in CDKN1B ubiquitination and subsequent proteasome degradation [33, 34]. It has been reported in breast cancer, lung cancer, ovarian carcinoma, and multiple myeloma [35–38] that the overexpressed CKS1B resulted in the tumor progression by promoting the degradation of p27Kip1 and patients with high CKS1B expression presented poor prognosis [39, 40]. These knowledge remarks points out the desirability of probing into pathobiology of CKS1B in OS.

In addition to exploring the expression and clinical significance of CKS1B, analysis of immune infiltration and single-cell RNA-seq (scRNA-seq) is applied for they are powerful technique to unfold content of biological mechanism [41, 42]. The composition of OS microenvironment contains abundant activated fibroblasts, neovascularization, infiltrated immune cells, and extracellular matrix components which requires pertinent processing [43, 44]. Traditional bulk RNA-seq has no way to obtain the heterogeneity of tumor cell clusters while scRNA-seq determines mRNA at cell level and comprehensively describes the complex situation of tumor microenvironment [45]. In summary, this study intends to evaluate the expression and pathological significance of CKS1B in OS through integrated analysis including IHC, microarray, and high-throughput datasets of public database, and the role of CKS1B serving as in OS TME is investigated through immune infiltration and data mining of scRNA-seq.

## 2. Patients and Methods

### 2.1. Verification of CKS1B Protein Expression in OS Tissues

Paraffin embedded tissue specimens and clinicopathological data were gained from OS patients who came to the Department of Orthopedic of Foshan Hospital of traditional Chinese medicine and were diagnosed from January 1, 2008 to June 1, 2021. Inclusion criteria of surgical specimen were as follows: ① The patient was diagnosed as OS by operation and pathology. ② Paraffin embedded OS tissue specimens are carefully preserved and have complete medical records. ③ Treatment including radiotherapy, interventional therapy, and medical therapy was not exerted.

CKS1B protein was detected by two-step method of Dako EnVision. Tumor tissue was sliced into sections with thickness of 4 *μ*m, and after dewaxing in xylene, alcohol hydration, PBS cleaning, and antigen repairing, I antibody was added. Then, II antibody was added after incubation at 4°C for 10 hours. Afterwards, sections were placed in a wet box and incubated at 37°C for 30 minutes. After PBS was washed again, DAB kit was used for color development, and hematoxylin was conducted to counterstain the nucleus. Finally, neutral gum was added to cover the wave plate seal.

The results were reviewed by two experienced pathologists. CKS1B protein was localized in the nucleus, showing brownish yellow particles, and brownish yellow cells signified positive expression. Under the 400× magnification, the IHC sections of each patient were randomly evaluated for 5–10 fields with dense and nonrepetitive cells. At least 100 cells were counted, and the percentage of positive cells was recorded. The score was measured according to the following criteria: proportion of positive cells in the total cells was less than 25% (1); positive cells accounted for 25%–50% of the total cells (2); positive cells accounted for 50%–75% of the total cells (3); positive cells accounted for more than 75% of the total cells (4). According to the staining intensity of positive tumor cells in each section, the score was cells that were not stained (0); cells were stained light yellow (1); cells were stained brown yellow (2); cells were stained brown (3). The final score of each OS section was obtained by multiplying the positive cell percentage score and staining intensity score divided into four groups: negative (−; 0), weak (+; 1–4), moderate (++; 5–8), and strong (+++; 9–12).

### 2.2. Differential Expression and Clinical Significance of CKS1B between OS and Nontumor Samples

3 pairs of OS and nontumor specimen were collected from OS patients who admitted at the Department of Orthopedic Surgery of The First Affiliated Hospital of Guangxi Medical University from 10 October, 2015 to 18 December, 2017. The tumors and nontumor tissues were surgically excised with the consent of patients. The comparative analysis of mRNA expression in these 6 cases was conducted with microarray technology provided by Shanghai Kangcheng Biological Company.

The datasets were gathered by searching GEO (http://www.ncbi.NLM.NIH.Gov/GEO/), ArrayExpress (https://www.ebi.Ac.uk/ArrayExpress/), and literature database, while gene expression and diagnostic data true positive, false positive, negative true, negative false, and true negative (TP, FP, FN, and TN) of CKS1B in OS and nontumor tissues were extracted. The expression of CKS1B in each study and the diagnostic ability were shown via violin diagram and receiver operating characteristic (ROC) curve. To further illustrate the expression level of CKS1B in OS and its ability of distinguishing OS tissues, integrated analysis was applied to evaluate public data and microarray data. Standard mean deviation (SMD) and 95% confidence interval were calculated. *I*^2^ > 50% and *P* value <0.05 indicate significant heterogeneity while SMD should be calculated by Random model; otherwise fixed model should be conducted. Begg's test was performed to evaluate the publication bias of the included studies. Finally, the diagnostic accuracy's test was employed to comprehensively judge the diagnostic ability of CKS1B mRNA via calculating the TP, FP, FN, and TN displaying in the form of Area under the Curve (AUC) of summary receiver operating characteristic (sROC). The relationship between CKS1B mRNA and related clinical parameters was analyzed and Kaplan Meier (KM) plotter was conducted to reveal the effect of CKS1B mRNA on overall survival of OS patients.

### 2.3. Immune Infiltration and Checkpoint Analysis Reflecting the Role of CKS1B in Tumor Microenvironment (TME) of OS

The composition and abundance of immune cells in TME have a great impact on tumor progression and the effect of immunotherapy. Timer 2.0 (http://timer.cistrome.org/) is an open interactive web service database that can systematically evaluate tumor infiltrating immune cells and evaluate the relationship between targeted gene expression and immune cell. In this study, R package immunedeconv [46] integrating timer, XCELL, MCP, Cell type Identification by Estimating Relative Subsets of RNA Transcripts (CIBERSORT), EPIC, and QUANTISEQ were applied to reveal the immune cells distribution of 88 OS patients, and the correlation between CKS1B expression and tumor infiltrating immune cells (TICs) was explored. Concurrently, association between expression of immune checkpoint gene and CKS1B was calculated.

### 2.4. The Expression of CKS1B in OS Cells from the View of Single-Cell Level

110869 single-cell transcriptomes in GSE152048 from 11 OS patients were obtained via GEO. The raw unique molecular identifier (UMI) counts data of cells were analyzed R package Seurat [47] which was performed to correct deviation factors and generated unbiased expression matrix. Simultaneously, uniform manifold approximation and projection (UMAP) was applied to reduce the dimension of the data, and expression distribution was displayed via two dimensions (UMAP1, UMAP2) to describe the relationship between various types of cells. Marker genes among different cell clusters were calculated by Wilcoxon rank sum test while the screening criteria were log2 fold change > 1 and *P* value <0.05. The marker genes of each cluster were compared with information provided by Cell Marker database, concurrently consulting with the cell-specific genes reported in the literature, to annotate the cell cluster and investigate the heterogeneity of CKS1B expression in each cell community of TME.

### 2.5. Functional Analysis of Marker Genes of Cell Clusters Where CKS1B is Upregulated in

Analysis of Gene Ontology (GO) enrichment is mainly described from the following three aspects: biological process (BP), molecular function (MF), and cellular component (CC) associated with biological phenotype. The annotation from GO database was downloaded to classify genes differentially expressed and *P* value was corrected by false discovery rate (FDR). Biological pathways analysis is based on Kyoto Encyclopedia of Genes and Genomes (KEGG) database. The differential genes are annotated according to KEGG database, and conspicuousness level of pathway was evaluated by Fisher's exact test to screen the pathways with remarkable gene enrichment. The significance was judged by the *P* value <0.05 after FDR correction.

### 2.6. Deduction of Gene Regulatory Networks and Related Cell States from Single-Cell RNA Data

Single-Cell Regulatory Network Inference and Clustering (SCENIC) [48] is a tool to reconstruct gene regulatory networks and identify stable cell states of scRNA-seq data. The gene regulatory network (GRN) is inferred based on coexpression and DNA motif analysis, and then the network activity is investigated in each cell to identify the cell state. The process of SCENIC mainly includes the following 4 steps:

  Step 1: GRNboost2 algorithm was applied to identify and screen the coexpression genes with transcription factors (TFs).
  Step 2: potential direct binding targets of coexpression module were sifted based on DNA motif analysis.
  Step 3: the transcriptional activity of each regulon was measured by AUCell algorithm to determine the regulation intensity of TFs on single cell.
  Step 4: cells are classified according to GRN activity

In addition, the information of gene-motif rankings and annotation of motifs to transcription factors were download from cisTarget (https://resources.aertslab.org/cistarget/) where motifs in the gene promoter and 20 kB (±10 kB) around the transcription start site (TSS) were integrated. For a separate regulon, AUCell scores among all cells are compared to identify cells that have more prominent regulon activity.

### 2.7. Construction of NMF Molecular Classification Analysis Based on CKS1B Coexpression Genes

Weighted Genes Correlation Network Analysis (WGCNA) was used to analyze the expression correlation coefficient between CKS1B mRNA and other genes in TARGET-OS expression matrix to obtain the coexpression genes that may participate in the same biological process with CKS1B.

In recent years, nonnegative matrix factorization (NMF) has been widely used in the field of bioinformatics with the greatest advantage for the ability of identifying the local characteristics of data and quantitatively describing the potential and additive nonlinear combination relationship between local and whole。. In this study, NMF R package was conducted for clustering molecular classification analysis. The clustering number *K* value was selected as 2∼10 while *K* value with the best stability was selected according to the clustering effect. The correlation between molecular classification based on NMF model and tumor metastasis as well as prognosis was analyzed. What is more, gene set variation analysis (GSVA) was used to calculate the pathway phenotype of each cluster.

### 2.8. Statistical Analysis

The statistical analysis in the study was completed by SPSS 24.0 (SPSS Inc., Chicago, IL, USA), R version 4.0.3 (https://www.r-project.org/), GraphPad prism 7.0, Stata 14.0 (http://www.stata.com). The expression level of CKS1B between OS and nontumor groups was analyzed. For the comparison between two continuous variables, the normality test Kolmogorov-Smirnov was performed first, such as normal distribution of data (*α* > 0.10), homogeneity test of variance, the two sample *t*-test of homogeneous square difference (*P* > 0.10), and the approximate *t*-test of uneven variance. If any group of data is biased, Mann-Whitney *U* test is performed. The results are expressed as mean ± standard deviation. Log-rank test was utilized to determine whether these survival curves could reveal the difference in prognosis of patients of different groups. Fisher's exact test was conducted to test the results of biological function and pathway enrichment analysis. *P* < 0.05 was regarded as the criterion to determine whether the result was statistically significant.

## 3. Results

### 3.1. IHC Verified the Upregulated Expression of CKS1B Protein in OS Tissues

**Figure 1:**
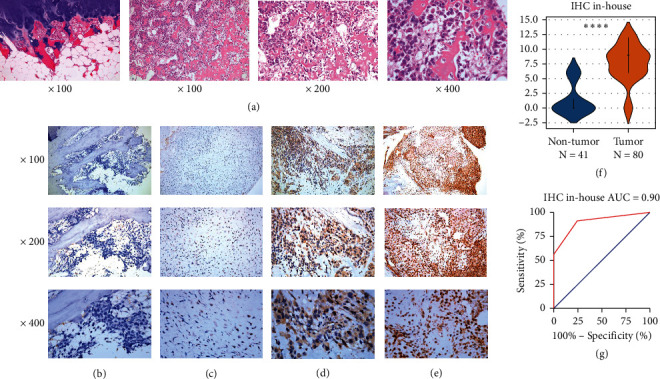
Hematoxylin-eosin (HE) staining and immunohistochemistry (IHC) staining of CKS1B in osteosarcoma (OS) and nontumor tissues. (a) HE staining of CKS1B in nontumor tissues (100×) and OS tissues (100×, 200×, 400×); (b) negative IHC staining of CKS1B in nontumor tissues (100×, 200×, 400×); (c) weak positive IHC staining of CKS1B in OS tissues (100×, 200×, 400×); (d) moderate positive IHC staining of CKS1B in OS tissues (100×, 200×, 400×); (e) strong positive IHC staining of CKS1B in OS tissues (100×, 200×, 400×); (f) violin-plot of IHC staining displayed expression analysis of CKS1B; (g) the receiver operator characteristic (ROC) curve of IHC staining displayed expression analysis of CKS1B (“^*∗∗∗∗*^” means *P* < 0.001).

The expression of CKS1B was mainly localized in the nucleus of tumor cells. The CKS1B staining results of 80 OS tissues and 41 nontumor tissues are shown in Figure 1. Among 80 OS tissues, 7 were negative, 28 were positive, and 45 were strongly positive (Figure 1). In 41 nontumor tissues, only 10 cases were positive, and 31 cases were negative. There was significant difference in the expression of CKS1B between OS and nontumor tissues (*P* < 0.001, Figure 1(f)). The area under the characteristic curve of subjects diagnosed with OS was 0.90 (Figure 1(g)).

### 3.2. Upregulated Expression of CKS1B mRNA in OS Verified by Microarray and Public Datasets

**Figure 2:**
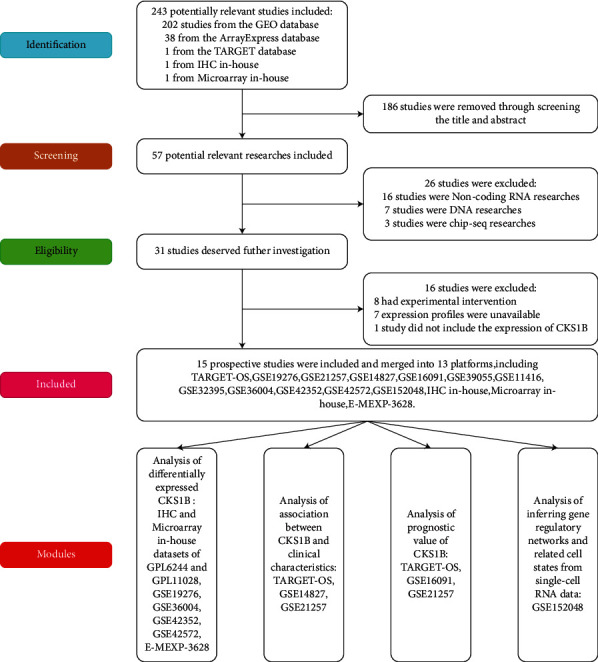
Flow chart of data collection for this study reflecting the process from the retrieval of public datasets to the final inclusion of qualified studies.

**Figure 3:**
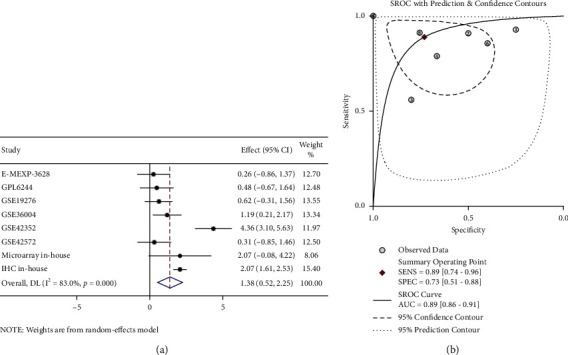
Pooled standard mean deviation (SMD) forest plot and summarized receiver operating characteristic (sROC) curves of CKS1B in osteosarcoma (OS) for in-house tissue microarray, external microarrays, and IHC staining. (a) Pooled SMD forest plot reflected overexpression of CKS1B in OS. (b) sROC curve reflected discriminatory ability of CKS1B expression of distinguishing OS from nontumor tissue.

Analysis of microarray in-house showed that expression of CKS1B mRNA in OS tissues had an elevated trend (Figure S1(a)) and ROC curve (AUC = 1.00, Figure S2(a)) indicated that it possessed a strong ability of distinguishing OS from nontumor samples. The outcome of tissue microarray combined with public datasets showed that there were 8 studies including 114 OS and 39 nontumor samples (Figure 2, Table 1). The integrated analysis was applied to verify CKS1B expression in OS and results of heterogeneity test showed that *I*^2^ = 81.5% (*P* < 0.001; see Figure S3(a)), suggesting that there was great heterogeneity; thus the random effect model was selected. The analysis results showed that CKS1B was significantly overexpressed in OS (SMD = 1.27, 95% CI: 0.23–2.30, and *P* < 0.05; see Figure S3(a)). Funnel plot revealed that there was no significant publication judged by Begg's test in the included studies (*P* = 0.23; see Figure S3(b)). The outcome of integration analysis of diagnostic accuracy demonstrated that the summary sensitivity and specificity were 0.89 and 0.74, respectively (Figure S3(c)). The AUC of sROC is 0.90 (95% CI: 0.87–0.93; see Figure S3(d)) calculated by fourfold table. Concurrently, the overexpression and diagnostic ability of CKS1B in OS were further confirmed through comprehensive curves of SMD and sROC combined with IHC data (SMD = 1.38, 95% CI: 0.52–2.25, AUC = 0.89, and 95% CI: 0.86–0.91; see Figure 3).

### 3.3. Pathological and Clinical Significance of CKS1B Expression in OS

The analysis of CKS1B expression and clinical parameters demonstrated that there was no significant difference in CKS1B mRNA expression between subgroups of chemotherapy sensitivity, OS recurrence, and metastasis (Figure S4). The integration of CKS1B expression on OS patients with metastasis presented a nonsignificant upward trend (SMD = 0.17 and 95% CI: −0.17–0.50; see Figure S5(a)). Kaplan Meier curve of 3 datasets showed that prognostic value of CKS1B expression in OS patients was insignificant (HR = 0.94 and 95% CI: 0.57, 1.55; see Figure S5(b)). TIDE method (http://tide.dfci.harvard.edu/login/) was conducted to evaluate the predictive of CKS1B in immunotherapy response while the result is negative (Figure S4(e)).

### 3.4. CKS1B Influenced CD4 Th2 Cells and Neutrophils in TME

**Figure 4:**
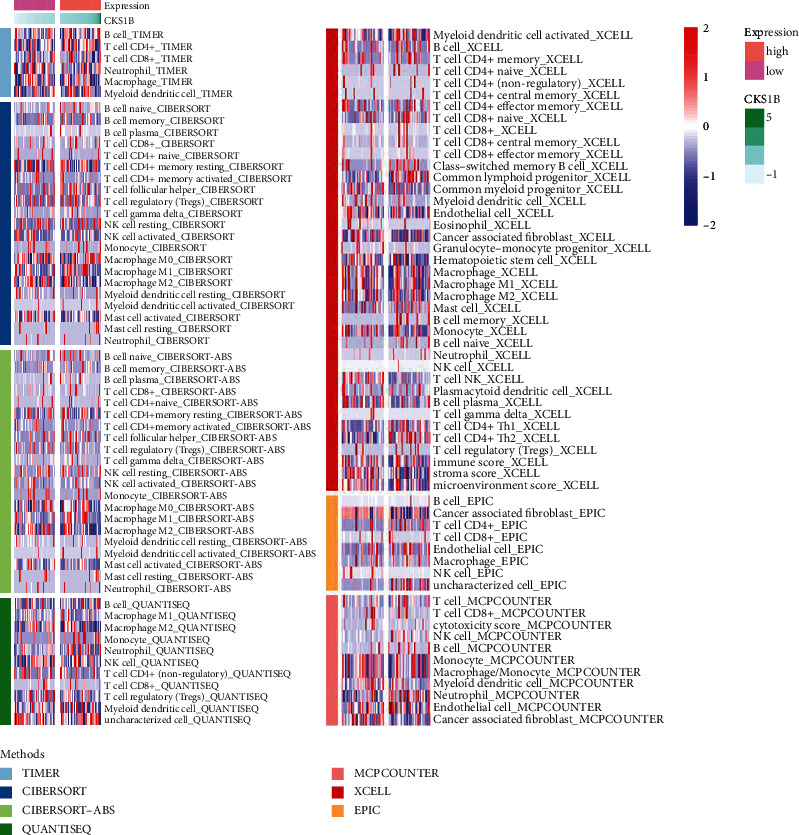
Heatmap for immune infiltration based on cell type identification by estimating relative subsets of RNA transcripts (CIBERSORT), ESTIMATE, MCP counter, single sample gene set enrichment analysis (ssGSEA), and TIMER algorithms among patients of TARGET-OS.

Patients of TARGET-OS project were divided into two groups via median of CKS1B expression conducted as threshold. Based on algorithms including Cell type Identification by Estimating Relative Subsets of RNA Transcripts (CIBERSORT), ESTIMATE, MCP counter, and single sample gene set enrichment analysis (ssGSEA), the differences of immunomics and TME were investigated between two groups (Figure 4). The results showed that there was a remarkable positive correlation between CKS1B expression and T.cell.CD4.Th2_XCELL in TME as well as Neutrophil_QUANTISEQ (Figure S6). The correlation analysis between immune checkpoint genes and CKS1B did not display significant positive results (Figure S7).

### 3.5. CKS1B Was Upregulated in Proliferating Osteoblastic OS Cells

**Figure 5:**
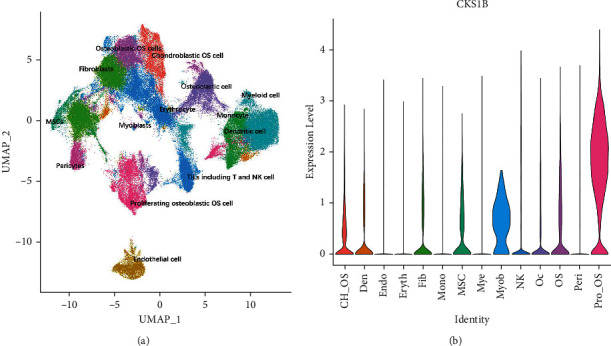
CKS1B was found to be upregulated in proliferating osteoblastic osteosarcoma (OS) cells. (a) Uniform manifold approximation and projection (UMAP) distribution of 14 annotated cell types. (b) Violin-plot displaying distribution of CKS1B in various types of cells. CH_OS: chondroblast OS cells, Den: CD1C + CD141+ dendritic cells, Eryth: erythrocyte, Fib: fibroblasts, Mono: monocyte, MSC: mesenchymal stem cells, Mye: myeloid cells, Myob: myoblasts, NK: TILs including T and NK cells, Oc: osteoclastic cells, OS: osteoblastic OS cells, Peri: pericytes, and Pro_OS: proliferating osteoblastic OS cells.

**Figure 6:**
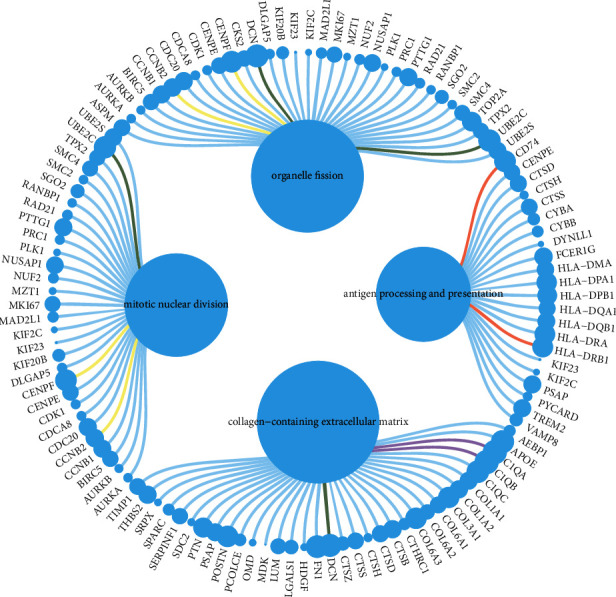
Enrichment analysis of signal pathway and function of differentially expressed genes of proliferating osteoblastic osteosarcoma (OS) cells where CKS1B is overexpressed.

The violin diagram represented the distribution of gene numbers, mRNA counts, and proportion of mitochondrial genes in the dataset. Invalid cells were filtered out while range of gene number of single cells is 500–4000, and the proportion of mitochondrial genes is 0.1 (Figures S9(a) and S9(b)). After quality control of the single-cell expression matrix of 11 OS patients, algorithm harmony was conducted to correct the batch effect (Figure S8). UMAP commendably reflected the continuity and organization of differentiation between cell clusters while umap1 and umap2 represent the spatial localization of cells (Figure S9(c)). The method of unsupervised clustering was applied to determine the cells as 37 different clusters. The distribution and spatial position of each cell cluster on UMAP diagram are shown in Figure S9(d). Through analysis of differential expression, highly expressed genes specific to each cell cluster were obtained. Consulting top 30 upregulated genes along with marker genes stored in Cell Marker database and the cell-specific marker genes reported in the literatures, 37 cell clusters were annotated as 14 cell types (myeloid cells, fibroblasts, osteoblastic OS cells, pericytes, TILs including T and NK cells, chondroblast OS cells, osteoclastic cells, monocyte, proliferating osteoblastic OS cells, mesenchymal stem cells, CD1C^+^CD141^+^ dendritic cells, endothelial cells, myoblasts, and erythrocyte; see Figure 5(a)) while CKS1B was obviously overexpressed in proliferating osteoblastic OS cells (Figure S9(e) and Figure 5(b)). In addition, marker genes significantly overexpressed in proliferating osteoblastic OS cells are enriched in biological functions and pathways such as organelle fission, mitotic nuclear division, antigen processing and presentation, and collagen-containing extracellular matrix (Figure 6).

### 3.6. Regulon Activity of Proliferating Osteoblastic OS Cells

**Figure 7:**
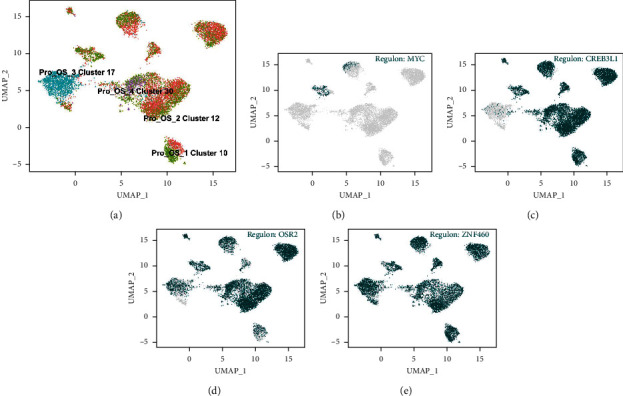
Regulon activity of proliferating osteoblastic osteosarcoma (OS) cells. (a) UMAP map of 4 clusters of proliferating osteoblastic OS cells based on regulon activity. (b) Binarized regulon activity scores for MYC regulon potentially regulated CKS1B on uniform manifold approximation and projection (UMAP) map (dark green dots). (c) Binarized regulon activity scores for top regulon CREB3L1 on UMAP map (dark green dots). (d) Binarized regulon activity scores for top regulon OSR2 on UMAP map (dark green dots). (e) Binarized regulon activity scores for top regulon ZNF460 on UMAP map (dark green dots).

**Figure 8:**
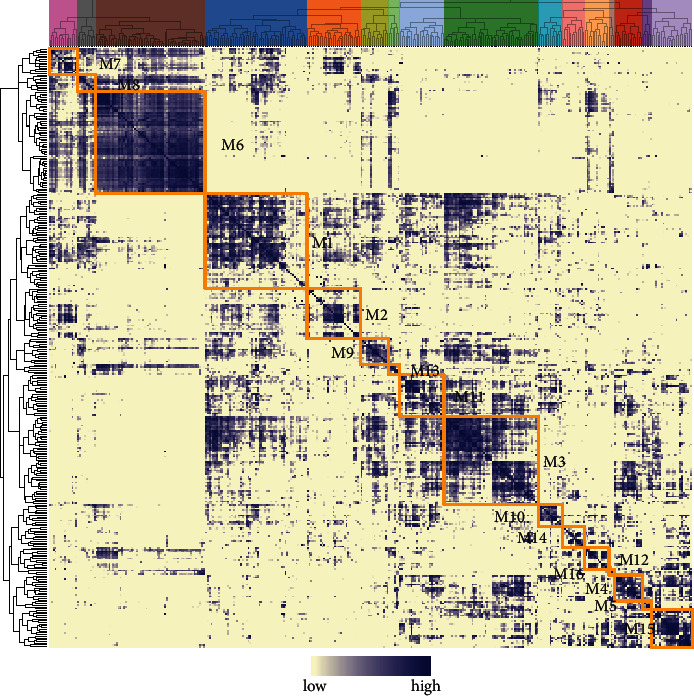
Heatmap of hierarchically clustered connection specificity index of regulon and 16 regulon modules were discerned.

The potential targets of TFS were identified based on the coexpression analysis of TFs and genes in the matrix. Then, motif enrichment analysis was carried out on the coexpression modules referring to the cisTarget database, and 347 motif modules with definite upstream regulators and significant enrichment were retained and defined as regulon. Afterwards, based on the expression genes attached to regulon, AUCell algorithm was conducted to evaluate the AUC of regulon activity in the cells. According to values of AUC, UMAP was performed on proliferating osteoblastic OS cells and homologous regulon activity distribution of cluster 10, cluster 12, and cluster 30 was discovered while cluster 17 was obviously separated (Figure 7(a)), which suggested that proliferating osteoblastic OS cells can be divided into subset for the disparate cytology function. What is more, CKS1B was detected to be contained in MYC Regulons (Table 2, Figure 7(b)). Finally, the regulons were hierarchically clustered by connection specificity index calculated from AUC and it was found that the CAMP Responsive Element Binding Protein 3 Like 1 (CREB3L1), Odd-Skipped Related Transcription Factor 2 (OSR2), and Zinc Finger Protein 460 (ZNF460) exert the most powerful regulation in proliferating osteoblastic OS cells (Figures 7(c)–7(e)) and 16 regulon modules were determined (Figure 8). Among them, CREB3L1 and OSR2 belonged to module 4 while MYC and ZNF460 were classified to module 2 and module 13.

### 3.7. The Coexpression Genes of CKS1B Enriched in Mitotic Cell Cycle Process

**Figure 9:**
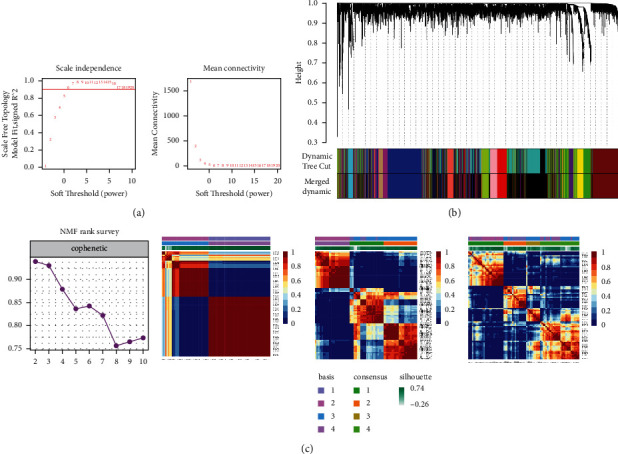
Weighted correlation network analysis (WGCNA) and nonnegative matrix factorization (NMF) analysis was conducted to explore coexpression genes of CKS1B. (a) The function of soft threshold parameter with scale-free fitting index and average connectivity. (b) Clustering system tree diagram of the combined gene matrix calculated by average hierarchical linkage clustering. The color squares below the tree diagram meant the modules cut by the dynamic tree. (c) Cophenetic line chart and heatmap of clustering pointed out *k* = 3.

**Figure 10:**
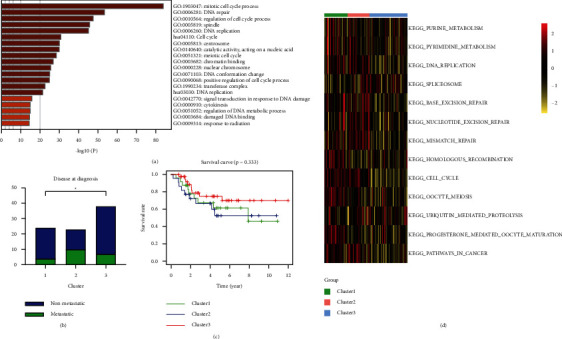
The molecular mechanism and clinic-pathological significance of NMF molecular type in TARGET-OS. (a) Gene Ontology (GO) and Kyoto Encyclopedia of Genes and Genomes (KEGG) enrichment analysis for module genes coexpressed with CKS1B. (b) Relationship between clusters and OS metastasis. (c) 3 Clusters showed prognostic differences of overall survival. (d) Gene set variation analysis (GSVA) of 3 clusters.

The sample system tree was drawn by analyzing the expression matrix of TARGET-OS containing 88 OS patients and soft threshold is set as the minimum integer value when the fitting coefficient *R*^2^ reaches 0.9 to ensure that coexpression network achieves the state of approximate scale-free network distribution. This study is based on *β* = 6 as soft threshold (Figure 9(a)). WGCNA combines highly similar modules through dynamic tree cut and hclust function to obtain cluster dendrogram (Figure 9(b)). Based on the degree of dissimilarity between genes, this study finally identifies 15 highly independent gene modules (excluding grey module). There are 790 genes in the Red module containing CKS1B, which are most significantly enriched in the mitotic cell cycle process (Figure 10(a)).

Based on the NMF model, the OS patients of TARGET-OS were typing at the molecular level, and the clustering stability was comprehensively judged while *k* = 3 presents the best stability (Figure 9(c)). Therefore, the OS samples were divided into three subgroups. *χ*^2^ test showed that there were significant differences in OS metastasis (Figure 10(b)). Simultaneously, by quantifying the pathway phenotypes of the three subtypes based on GSVA, it was found that the main characteristics of the subtype with the worst prognosis are high enrichment score of DNA replication, Mismatch Repair, and Cell Cycle pathway (Figures 10(c) and 10(d)), while the main characteristics of the subtype with the best prognosis are low enrichment score of the 3 pathways, which suggested that Mismatch Repair process may play a more important role in the deterioration of OS.

## 4. Discussion

This study analyzed the mRNA and protein expression of CKS1B in OS via multilevel evidence including IHC, microarray, and high-throughput data of public database. Meanwhile, effect of CKS1B exerted on OS with further resolution and specificity was analyzed by immune infiltration and data of scRNA-seq, which provided a completely new perspective for studying the related mechanisms of OS occurrence and metastasis. The following highlights should be clarified in our research: ① it is the first original research to explore CKS1B expression of OS in the world. Taking into account the low incidence rate of OS [59], sample size of IHC in-house (80 OS vs. 41 nontumor tissues) is a powerful confirmation for the upregulation of CKS1B protein in OS. ② Datasets embodied the expression trend of CKS1B coming from 6 countries in 4 containers, suggesting that the abnormal overexpression of CKS1B mRNA is universal in body, which is worthy for greater resources from institutions worldwide to investigate more intensive mechanism. ③ Via analysis of scRNA-seq and immune infiltration, the cell types of OS TME are more finely displayed while potential mechanism of CKS1B advancing OS metastasis is thoroughly explored.

Firstly, significant upregulation of CKS1B protein and mRNA level in OS was demonstrated, which is consistent with the findings of highly expressed CKS1B in nasopharyngeal, breast cancer, non-small-cell lung cancer, Burkitt lymphoma, and multiple myeloma [60–63]. Simultaneously, according to the expression Atlas of pan-cancer sorted in Gene Expression Profiling Interactive Analysis (GEPIA) (http://gepia.cancer-pku.cn/), it is found that CKS1B mRNA is obviously upregulated in epithelial and mesenchymal solid tumors as well as hematological malignancies, suggesting the status of cell cycle regulation in carcinogenesis. Proliferation of tumor cells caused by disorder of cell cycle regulation is the basic biological feature of malignant tumors while the main recognized mechanism is the dysfunction of monitoring points caused by ubiquitin proteasome pathway CKS1B participating in [64, 65]. Therefore, knowledge of cell cycle and the expression of related genes represented by CKS1B can ascertain the essence of tumor development. In addition, considering the positive application of cyclin-dependent kinase inhibitor proteins (CDKI) targeting downstream molecule of CKS1B in malignant tumors [66, 67], profound study of CKS1B might also provide a scientific basis for selecting specific drug targets.

Then, there was no significant correlation discovered between CKS1B expression of traditional high-throughput data and clinical traits comprised of drug sensitivity, recurrence, metastasis, and prognosis of OS patients, which is contrary with studies demonstrating that upregulated CKS1B mRNA expression is a risk factor bringing about the resistance of tumor cells for chemotherapy and poor prognosis in tumors including colorectal carcinoma, gastric carcinoma, follicular lymphoma, and ovarian carcinoma [68–71]. The bias of the results due to the restricted OS sample size of included studies and noise of the traditional high-throughput reflecting the mRNA average expression in complex composition of tissues might mainly lead to the heterogeneity between studies. Therefore, CKS1B expression in OS is more accurately defined in this study via analysis of scRNA-seq data and immune infiltration. Through the annotation of 110869 single cells, it was found that TILs including T and NK cell (22032), myoid cell (17047), and fibroblasts (18338) occupy the highest proportion in OS TME, suggesting the existence of nontumor components which affects the specificity of judging biological function of CKS1B by measurement of traditional high-throughput. More importantly, it was displayed that the CKS1B was upregulated in OS cells with high expression of DNA Topoisomerase II Alpha (TOP2A), Proliferating Cell Nuclear Antigen (PCNA), and Marker of Proliferation Ki-67 (MKI-67), which are closely related to cell promotion and apoptosis with specific expression status during cell division [72, 73]. What is more, the 3 genes extensively served as indexes to determine activity of cell proliferation to evaluate invasive biological process of tumor [74, 75]. These findings suggest that CKS1B may exert a stimulative effect on progression of OS.

Afterwards, enrichment analysis was conducted on specifically and differentially expressed genes of proliferating osteoblastic OS cell, and it was found that these genes were significantly enriched in functions and pathways containing antigen processing and presentation, organelle fission, and mitotic nuclear division and containing extracellular matrix. At present, mitosis-related genes have been confirmed to concern tumor progression in a few studies. Centromere Protein F (CENPF), a member of centromeric protein family, plays an important role in separation of chromosomes and assembly of spindle during cell division [76]. A study consists of 295 breast cancer patients demonstrated that overall survival obviously correlated with the upregulation of CENPF [77]. Homologously, the research by Cao et al. proved that CENPF was overexpressed in nasopharyngeal carcinoma and correlated with the overall survival of patients [78]. Polo Like Kinase 1 (PLK1) is a key regulator in mitosis and cytokinesis while it is highly expressed in most human tumor cells [79, 80]. It was discovered that blocking the expression of PLK1 via siRNA can effectively inhibit the proliferation and induce the apoptosis of tumor cells [81, 82]. The invasion and metastasis of malignant tumor are the result of a series of complex and multistep interactions among tumor cells, host cells, and extracellular matrix [83, 84]. Some studies have found that the growth of OS cells mediated by Focal Adhesion pathway depends on the mechanical strength of extracellular matrix [85] and adhesion between tumor and normal cells, extracellular matrix, and the degradation of extracellular basement membrane have served as the prerequisites for malignant tumor invasion [86]. What is more, it is known that the failure of antigen presentation and processing function in the body will affect the efficacy of killing tumor cells regulated by CTLs, causing tumor cells in blood circulation to escape the monitoring of immune system and lay foundation for tumor metastasis [87, 88]. At the same time, the abnormal antigen presentation and processing also limit the application of antigen-presenting cell vaccines such as dendritic cells in OS [89, 90]. In summary, the results of enrichment analysis suggest that proliferating osteoblastic OS cells with high CKS1B expression may be a crucial factor in promoting OS metastasis.

Similarly, our results showed that there was a significant positive correlation between the CKS1B expression and infiltration level of CD4^+^ Th2 cells as well as neutrophils, which equally suggest that CKS1B is involved in the process of OS metastasis. The antitumor immune effect of body usually gives priority to Th1 mediated cellular immune response, but most tumor patients suffer Th1/Th2 drift which is characterized by the predominance of Th2 cytokine synthesis [91]. Gaur P discovered that the expression levels of Interleukins 4 (IL-4) and IL-10 and Transforming Growth Factor (TGF-*β*) in the central region of OSCC are closely related to the activity of Th2 cells, which results in the inhibition of immune function, local infiltration, and lymph node metastasis [92]. After being cocultured with OS-S180 cells, tumor associated macrophages (TAMs) differentiated into M2 phenotype, and the secretion level of Th1/Th2 cytokines transformed into dominant Th2 cytokine expression [93]. Compared with neutrophils in blood, tumor associated neutrophils (TANs) around tumor can produce more cytokines and promote tumor progression by ways of secreting Matrix Metallopeptidase 9 (MMP-9) to advance the degradation of extracellular matrix and release vascular endothelial growth [94, 95]. Several studies on breast cancer have found that T cells can regulate TANs and promote lung metastasis of breast cancer while neutrophils were found to be accumulated in the lungs before metastasis [96, 97]. A study on melanoma with lung metastasis suggested that TANs can assist encapsulated tumor cells in escaping immune surveillance and these tumor cells are more likely to metastasize than scattered tumor cells [98]. The results could support the view of CKS1B promoting OS metastasis from scRNA-seq analysis.

In addition to studying the expression and biological function of CKS1B, the construction of CKS1B related transcriptional regulation network in OS can provide ideas for exploring the pathogenesis of OS and reference for optimizing therapeutic drugs. At present, the research on regulation of CKS1B in tumors is limited to the aspects of miRNA. Mechanistic investigations demonstrated that the miR-197-mediated CKS1B/STAT3 axis was excavated exerting tumor progression regulated by various protooncogenes like BCL2 Apoptosis Regulator (BCL2), MYC, and Cyclin D1(CCND1) [62]. What is more, miR-1258 was revealed to downregulate CKS1B expression through binding to the 3′- Untranslated Regions (UTR) [99]. In recent years, the rapid expansion of scRNA-seq technology can appraise the differences between various cells around TME and exploited abundant information for the study of the internal regulation mechanism of the cells [100]. This study focused on proliferating osteoblast OS cells with significantly upregulated expression of CKS1B and utilizing SCENIC workflow; the potential regulatory effect of MYC on CKS1B was detected, which was consistent with Keller et al.'s study discovering that MYC suppresses cyclin-dependent kinase inhibitor (p27Kip1) expression, accelerates cell proliferation, and promotes tumorigenesis through its ability of selectively inducing CKS1 [70]. MYC protein is a TF involved in regulating cell growth and proliferation, cell cycle, and apoptosis while overexpressed MYC mRNA has been proved to be closely related to the recurrence and metastasis of OS [10, 101, 102]. Restraint of MYC activation might be of great significance to inhibit the proliferation of OS and achieve better clinical efficacy for OS patients [10, 103]. Simultaneously, in proliferating osteoblastic OS, CREB3L1, ZNF460, and OSR2 were identified as the most active TFs. CREB3L1 is mainly distributed on the endoplasmic reticulum of osteoblasts and astrocytes [104]. Additionally, CREB3L1 is a key effector downstream of unfolded protein response (UPR) pathway and inhibits cell proliferation and induces apoptosis by binding cis acting elements of tumor suppressor genes [105, 106]. OSR2 contains DNA binding C2H2 type zinc finger domains in the C-terminal half and plays an important role in cellular silence and promotion under epigenetic regulation [107, 108]. Zinc finger protein family served as important TFs that have been reported to be widely involved in critical biological activities, such as proliferation, metabolic regulation, and signal transduction [109, 110]. However, researches on ZNF460 are deficient and this study took the lead in proclaiming the correlation between ZNF460 and tumor biology. The key role of these TFs in OS needs further research.

This study further analyzed the NMF molecular classification of TARGET-OS cohort based on CKS1B coexpression genes. The OS patients were divided into three subtypes and *χ*^2^ test as well as survival analysis was conducted to judge the effect of classification. It was found that there were significant differences in OS metastasis among the three clusters. By quantifying the pathway phenotypes of the three clusters based on GSVA, it was found that the cluster with the worst prognosis was mainly characterized by high enrichment score of DNA replication, Mismatch Repair (MMR), and Cell Cycle pathway, while the best prognosis cluster presented low enrichment score of the 3 pathways. DNA replication in the cell cycle is closely regulated by complex network of intracellular and extracellular signal pathways [111], involving cell proliferation, differentiation, apoptosis, and so on [112, 113]. The short tandem DNA repeats on the genome are called microsatellite sequences, which are prone to mismatch in the process of DNA replication [114]. The MMR system mainly identifies and repairs the mismatched bases inserted in the process of DNA replication, adjusts the nucleotide sequence to the normal state, maintains the accuracy of DNA replication, and eliminates DNA damage [115]. And the repair function will be lost while mutation occurs in MMR system leading to abnormal expression of downstream target genes promoting neoplasia [116]. Jentzsch et al.'s study showed the prognostic value of DNA MMR protein expression as a marker of poor prognosis in OS patients [117]. In brief, our study suggests that, in CKS1B coexpression genes, the active MMR system in OS formation may be an important factor causing the deterioration of OS and poor prognosis of patients.

Although many encouraging findings were excavated in this study, current work still had some limitations. Our study emphasized the clinical significance of CKS1B in OS, but successive experiments in vitro and in vivo were still needed to further verify the biological role of CKS1B in OS. In addition, although this study employed various methods to detect the expression of CKS1B in OS, status of CKS1B in the peripheral blood of OS patients had not been explored; thus whether the expression of CKS1B in peripheral blood had homologous tendency with that in OS tissues and its pathology clinical significance remained clarified. In the meantime, there were few studies exploring the diagnostic value of CKS1B in other tumors. Therefore, intensive evidence is required for clinical application of CKS1B via collecting serum or plasma samples from OS patients in subsequent studies.

## 5. Conclusions

In summary, upregulation of CKS1B expression in OS tissue and cells was confirmed through multilevel evidence. What is more, specific overexpression of CKS1B on aggressive OS cells combined with the evolution of immune infiltration for CKS1B exerting influence on Th1/Th2 deviation and neutrophil polarization in TME of OS suggests the function of CKS1B promoting OS invasiveness. The present study on CKS1B in OS indicated a promising prospect for CKS1B as a biomarker and therapeutic target for OS.

## Figures and Tables

**Table 1:** Basic information for all included OS datasets.

Cohorts	Year	Country	Platform	OS sample	Nontumor sample	Type
Microarray in-house	2019	China	Arraystar Human LncRNA Microarray v4.0	3	3	Tissue
TARGET-OS	2019	USA	Illumina	88	0	Tissue
E-MEXP-3628	2012	Israel	HG-U133	14	4	Tissue
GSE19276 [49]	2010	Australia	GPL6848	44	5	Tissue
GSE21257 [50]	2011	Norway	GPL10295	53	0	Tissue
GSE14827 [51]	2010	Japan	GPL570	27	0	Tissue
GSE16091 [52]	2009	USA	GPL96	34	0	Tissue
GSE152048 [53]	2020	China	GPL24676	11	0	Tissue
GSE11416 [54]	2009	Canada	GPL6244	4	2	Cell line
GSE32395 [55]	2011	Germany	GPL6244	7	2	Cell line
GSE36004 [56]	2012	Norway	GPL6102	19	6	Cell line
GSE42352 [57]	2012	Norway	GPL10295	19	15	Cell line
GSE42572 [58]	2015	Norway	GPL13376	7	5	Cell line

**Table 2:** 3 types of MYC regulons potentially positive regulated CKS1B arranged in cisTarget databases.

TF	MotifID	AUC	NES	Motif similarity Qvalue	Orthologous identity	Context	Rank at max
MYC	dbcorrdb__NRF1__ENCSR000EHH_1__m1	0.039653	3.125732	0.00094	1	Weight > 75.0%	4827
MYC	dbcorrdb__NRF1__ENCSR000DZO_1__m1	0.039773	3.15208	0.000371	1	Weight > 75.0%	4645
MYC	dbcorrdb__NRF1__ENCSR000EHZ_1__m1	0.039476	3.0869	0.000519	1	Weight > 75.0%	4490

TF: transcription factor; AUC: Area under the Curve; NES: normalized enrichment score.

## Data Availability

The public data employed in the manuscript are stored in GDC (https://portal.gdc.cancer.gov/), GEO (https://www.ncbi.nim.nih.gov/geo/), and ArrayExpress (https://www.ebi.ac.uk/arrayexpress/).

## Supplementary Materials

Supplementary Materials Figure S1: violin-plot displayed CKS1B expression in osteosarcoma (OS) and nontumor samples from in-house tissue microarrays and external microarrays. Figure S2: the ability of CKS1B expression of distinguishing OS from nontumor tissues in each microarray dataset. Figure S3: integrative analysis of CKS1B expression in osteosarcoma (OS) of each microarray dataset. Figure S4: the clinic-pathological significance of CKS1B in osteosarcoma (OS). Figure S5: integrated analysis of the prognostic value of CKS1B reflecting osteosarcoma (OS) patients' occurring metastasis and survival condition. Figure S6: Pearson's correlation analysis showing association between CKS1B and immune cells. Figure S7: Pearson's correlation analysis showing association between CKS1B and immune checkpoint genes. Figure S8: the expression matrix of 11 samples osteosarcoma (OS) patients was integrated by R package harmony and it showed that 11 samples have strong fitting degree after eliminating batch effect. Figure S9: scRNA-seq analysis process of GSE152048 before cell annotation.
