# Supplementary figures and images for: The Clinical Significance and Potential Molecular Mechanism of Upregulated CDC28 Protein Kinase Regulatory Subunit 1B in Osteosarcoma
Source: J Oncol. 2021 Dec 10;2021:7228584. doi: 10.1155/2021/7228584 (PMC8683182; doi:10.1155/2021/7228584)

**Microarray in-house**

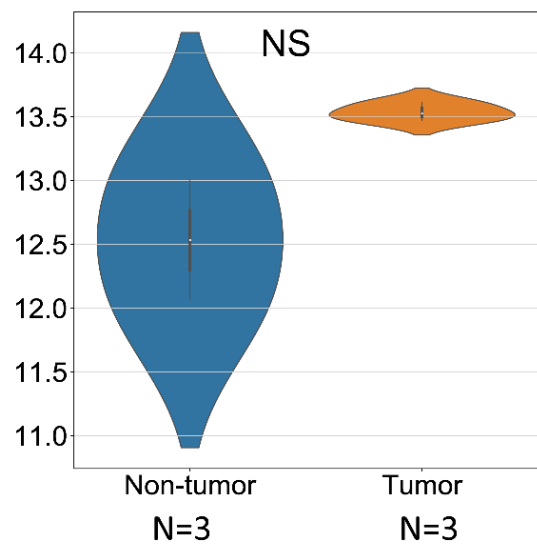

(a)

**E-MXEP-3628**

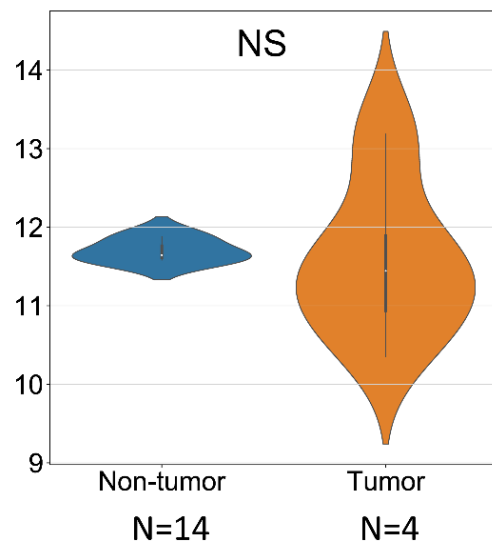

(b)

**GPL6244**

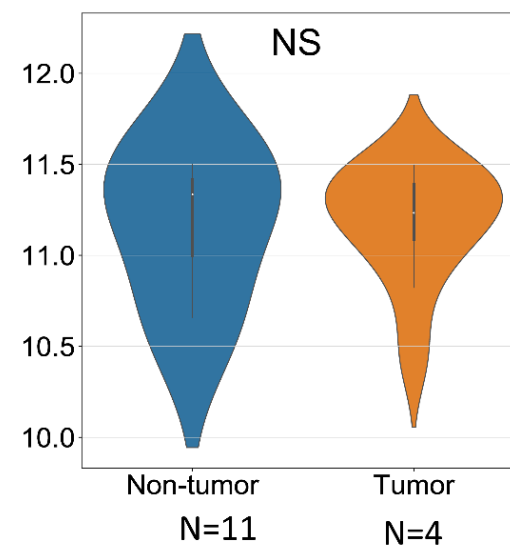

(c)

**GSE19276**

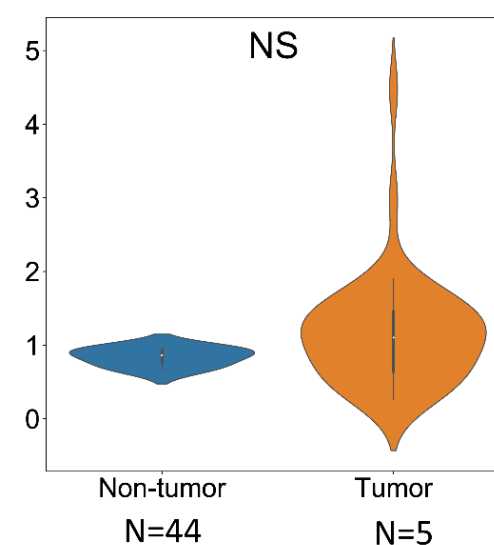

(d)

**GSE36004**

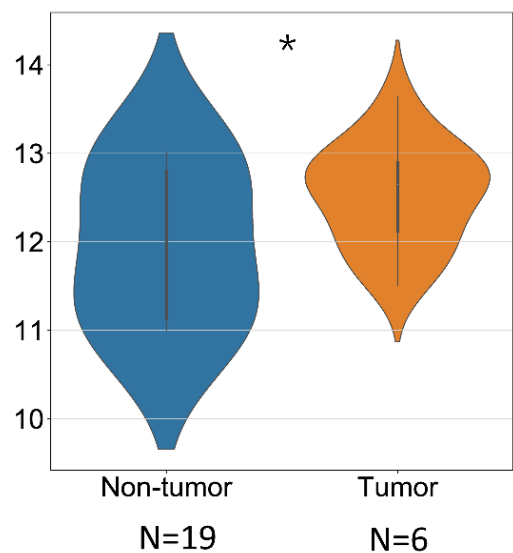

(e)

**GSE42352**

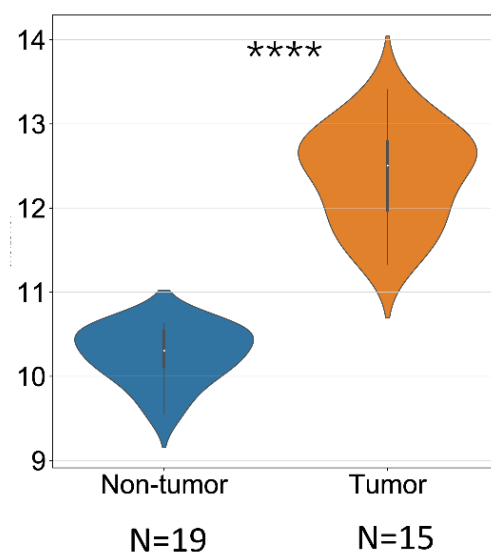

(f)

**GSE42572**

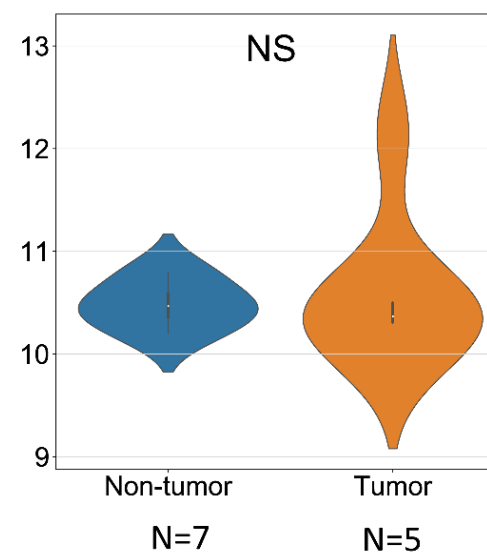

(g)

Supplement: Supplementary Materials — Figure S1: violin-plot displayed CKS1B expression in osteosarcoma (OS) and nontumor samples from in-house tissue microarrays and external microarrays. Figure S2: the ability of CKS1B expression of distinguishing OS from nontumor tissues in each microarray dataset. Figure S3: integrative analysis of CKS1B expression in osteosarcoma (OS) of each microarray dataset. Figure S4: the clinic-pathological significance of CKS1B in osteosarcoma (OS). Figure S5: integrated analysis of the prognostic value of CKS1B reflecting osteosarcoma (OS) patients' occurring metastasis and survival condition. Figure S6: Pearson's correlation analysis showing association between CKS1B and immune cells. Figure S7: Pearson's correlation analysis showing association between CKS1B and immune checkpoint genes. Figure S8: the expression matrix of 11 samples osteosarcoma (OS) patients was integrated by R package harmony and it showed that 11 samples have strong fitting degree after eliminating batch effect. Figure S9: scRNA-seq analysis process of GSE152048 before cell annotation. [file 7228584.f1.zip › 7228584.f1/Figure S1.pdf]

**Microarray in-house AUC=1.00**

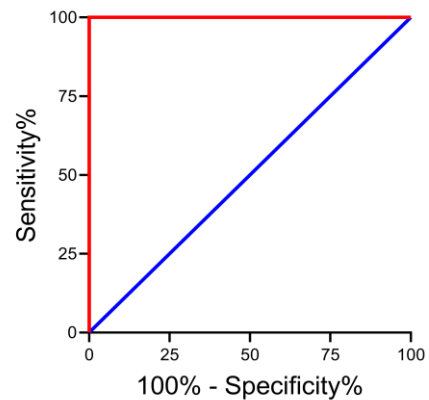

(a)

**E-MEXP-3628 AUC=0.53**

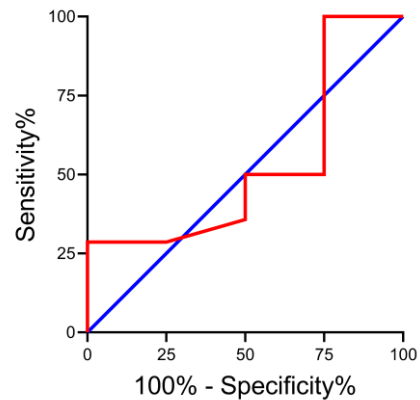

(b)

**GPL6244 AUC=0.57**

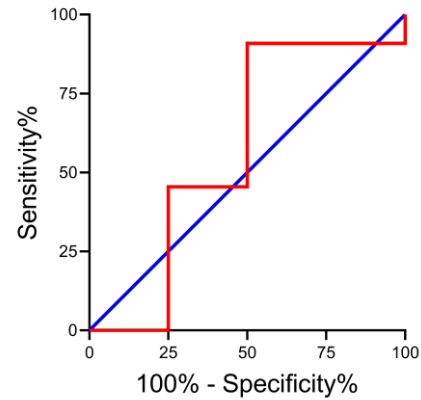

(c)

**GSE19276 AUC=0.73**

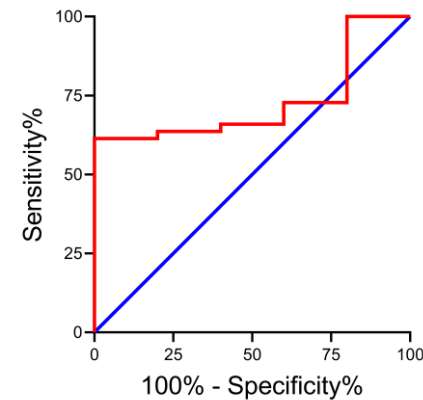

(d)

**GSE36004 AUC=0.71**

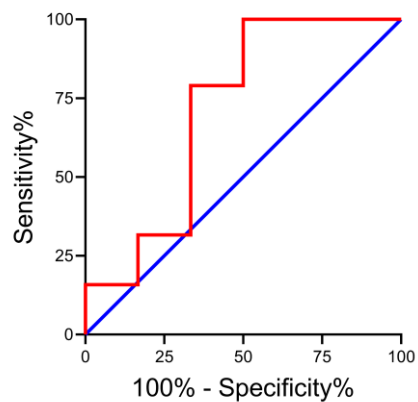

(e)

**GSE42352 AUC=1.00**

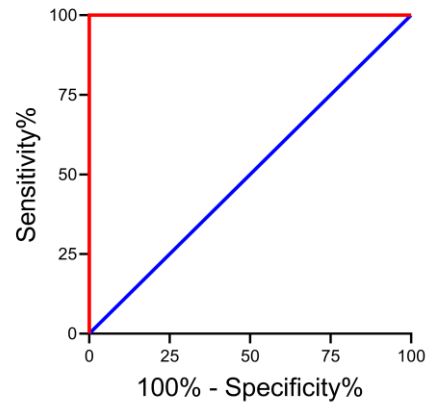

(f)

**GSE42572 AUC=0.51**

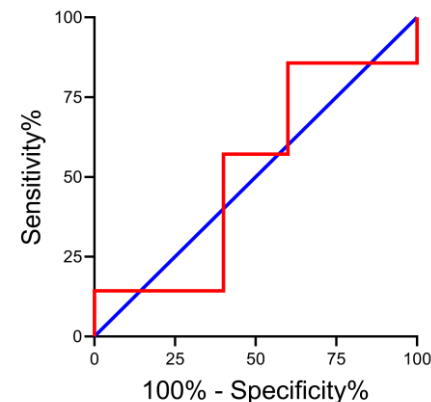

(g)

Supplement: Supplementary Materials — Figure S1: violin-plot displayed CKS1B expression in osteosarcoma (OS) and nontumor samples from in-house tissue microarrays and external microarrays. Figure S2: the ability of CKS1B expression of distinguishing OS from nontumor tissues in each microarray dataset. Figure S3: integrative analysis of CKS1B expression in osteosarcoma (OS) of each microarray dataset. Figure S4: the clinic-pathological significance of CKS1B in osteosarcoma (OS). Figure S5: integrated analysis of the prognostic value of CKS1B reflecting osteosarcoma (OS) patients' occurring metastasis and survival condition. Figure S6: Pearson's correlation analysis showing association between CKS1B and immune cells. Figure S7: Pearson's correlation analysis showing association between CKS1B and immune checkpoint genes. Figure S8: the expression matrix of 11 samples osteosarcoma (OS) patients was integrated by R package harmony and it showed that 11 samples have strong fitting degree after eliminating batch effect. Figure S9: scRNA-seq analysis process of GSE152048 before cell annotation. [file 7228584.f1.zip › 7228584.f1/Figure S2.pdf]

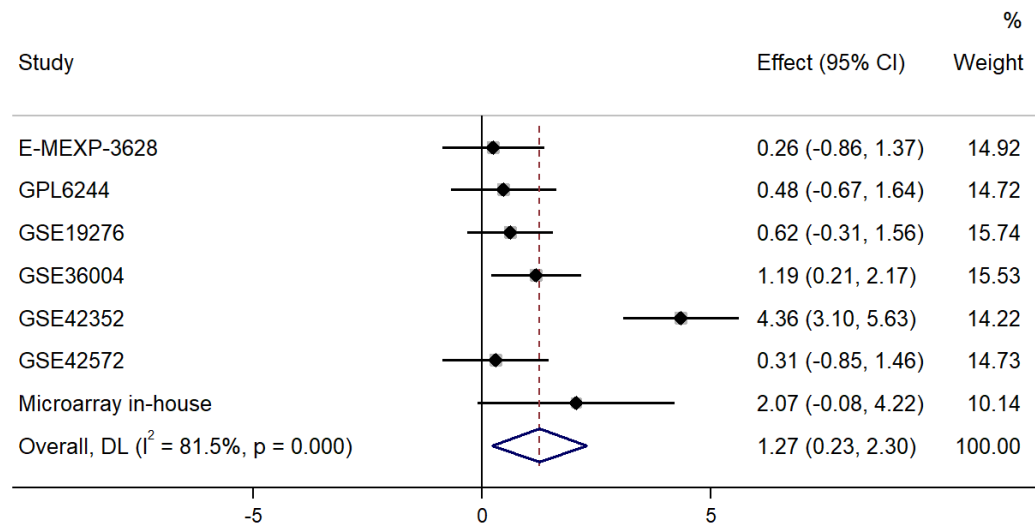

(a)

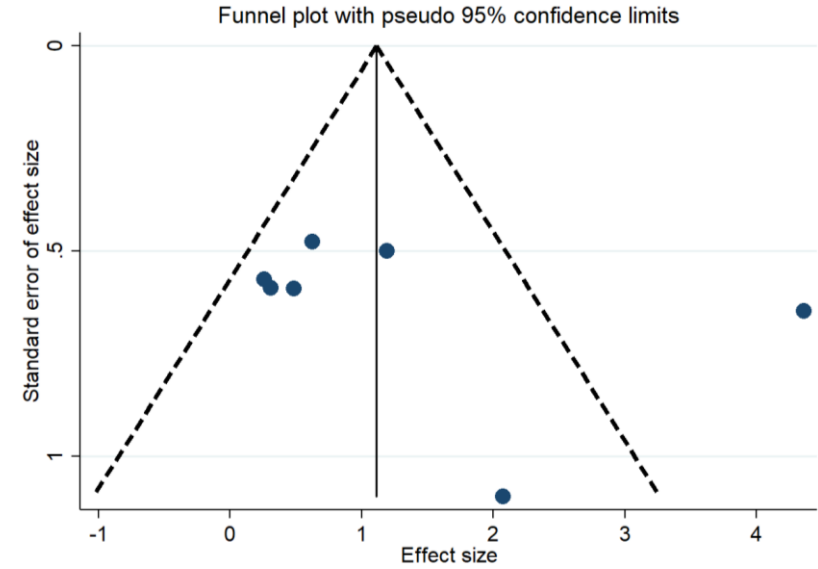

(b)

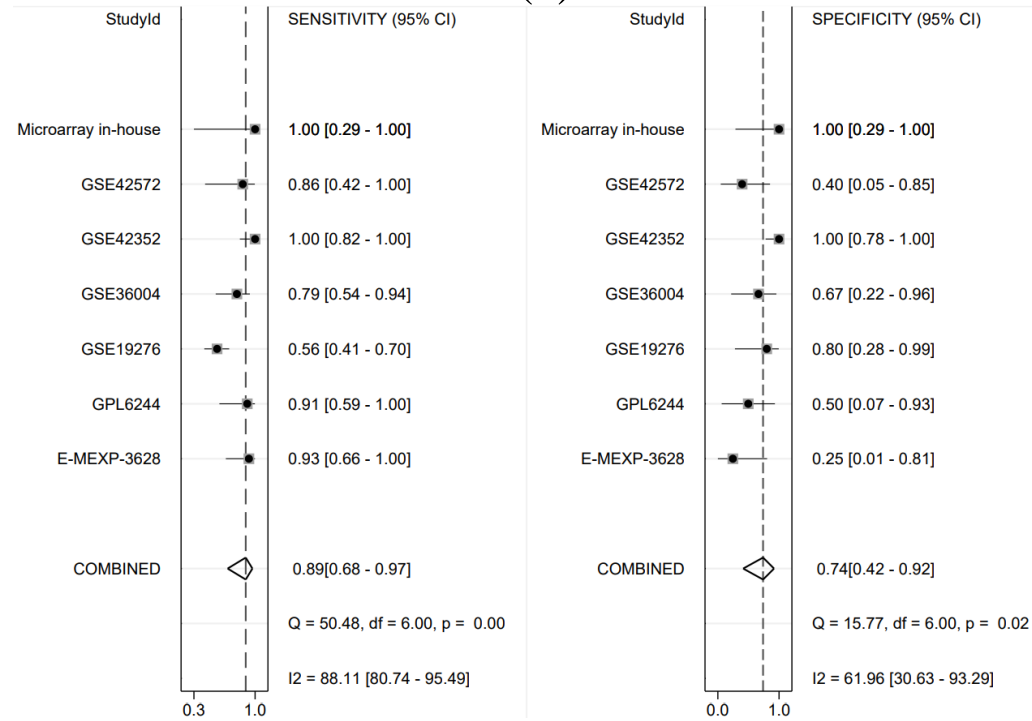

(c)

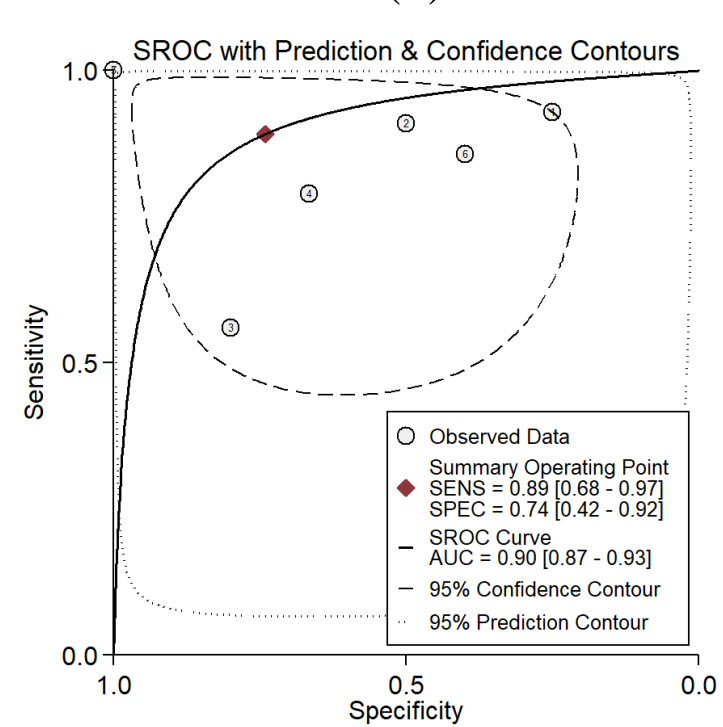

(d)

Supplement: Supplementary Materials — Figure S1: violin-plot displayed CKS1B expression in osteosarcoma (OS) and nontumor samples from in-house tissue microarrays and external microarrays. Figure S2: the ability of CKS1B expression of distinguishing OS from nontumor tissues in each microarray dataset. Figure S3: integrative analysis of CKS1B expression in osteosarcoma (OS) of each microarray dataset. Figure S4: the clinic-pathological significance of CKS1B in osteosarcoma (OS). Figure S5: integrated analysis of the prognostic value of CKS1B reflecting osteosarcoma (OS) patients' occurring metastasis and survival condition. Figure S6: Pearson's correlation analysis showing association between CKS1B and immune cells. Figure S7: Pearson's correlation analysis showing association between CKS1B and immune checkpoint genes. Figure S8: the expression matrix of 11 samples osteosarcoma (OS) patients was integrated by R package harmony and it showed that 11 samples have strong fitting degree after eliminating batch effect. Figure S9: scRNA-seq analysis process of GSE152048 before cell annotation. [file 7228584.f1.zip › 7228584.f1/Figure S3.pdf]

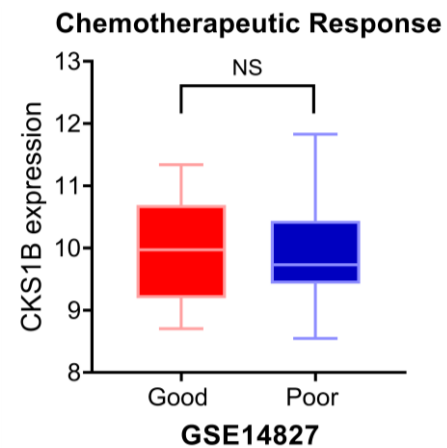

(a)

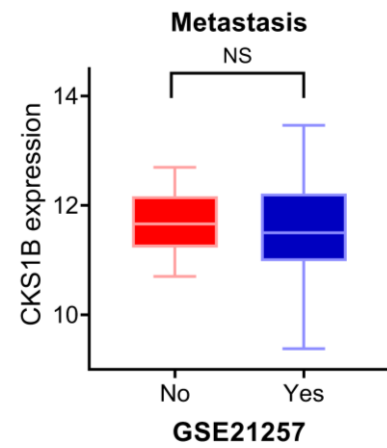

(b)

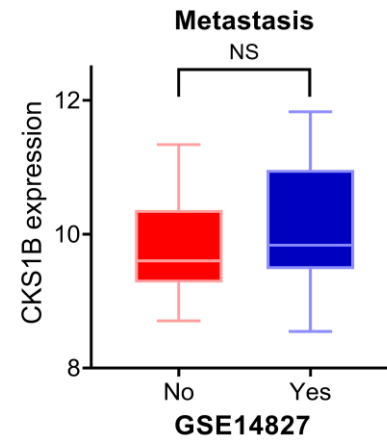

(c)

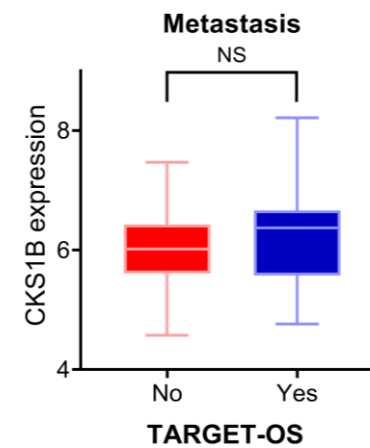

(d)

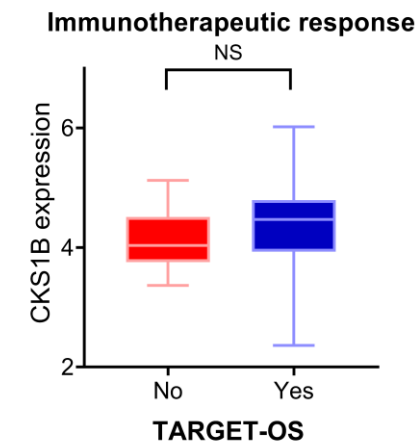

(e)

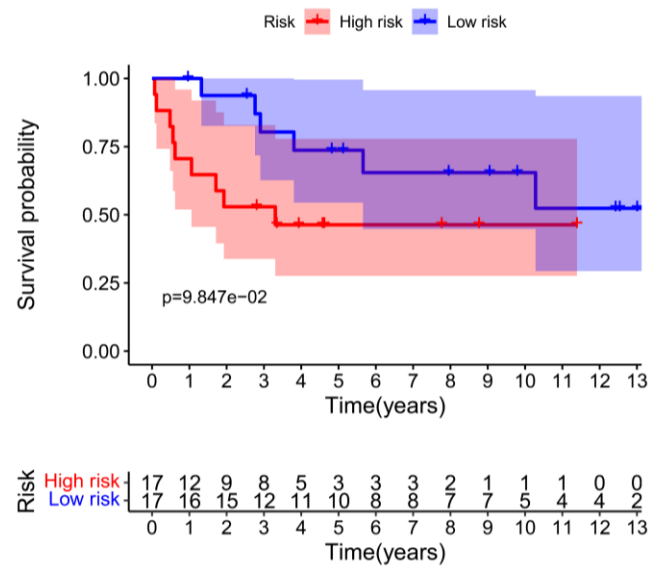

(f)

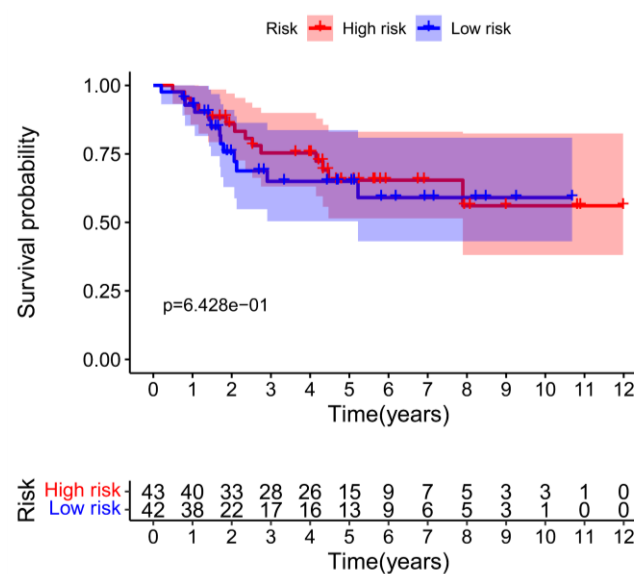

(g)

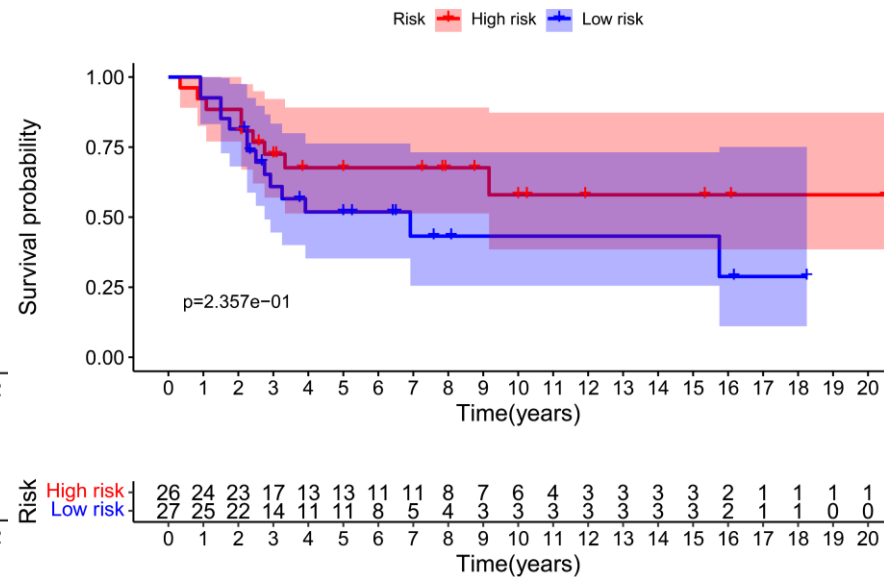

(h)

Supplement: Supplementary Materials — Figure S1: violin-plot displayed CKS1B expression in osteosarcoma (OS) and nontumor samples from in-house tissue microarrays and external microarrays. Figure S2: the ability of CKS1B expression of distinguishing OS from nontumor tissues in each microarray dataset. Figure S3: integrative analysis of CKS1B expression in osteosarcoma (OS) of each microarray dataset. Figure S4: the clinic-pathological significance of CKS1B in osteosarcoma (OS). Figure S5: integrated analysis of the prognostic value of CKS1B reflecting osteosarcoma (OS) patients' occurring metastasis and survival condition. Figure S6: Pearson's correlation analysis showing association between CKS1B and immune cells. Figure S7: Pearson's correlation analysis showing association between CKS1B and immune checkpoint genes. Figure S8: the expression matrix of 11 samples osteosarcoma (OS) patients was integrated by R package harmony and it showed that 11 samples have strong fitting degree after eliminating batch effect. Figure S9: scRNA-seq analysis process of GSE152048 before cell annotation. [file 7228584.f1.zip › 7228584.f1/Figure S4.pdf]

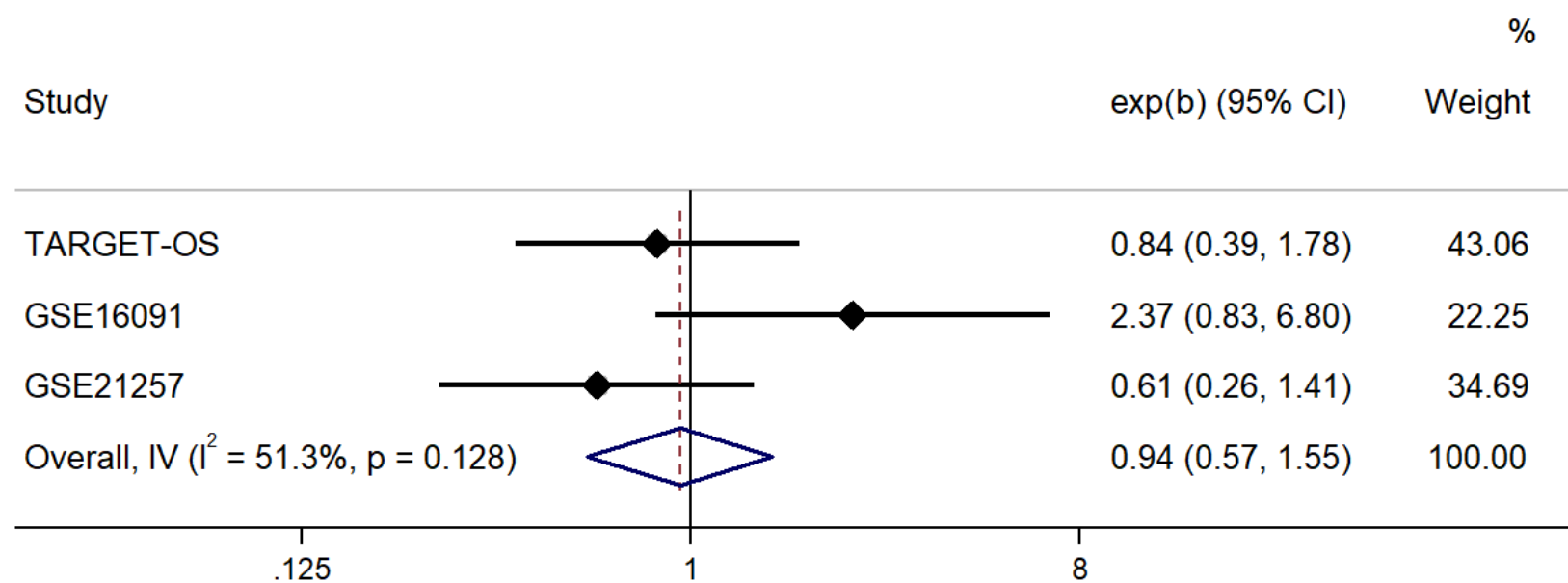

(a)

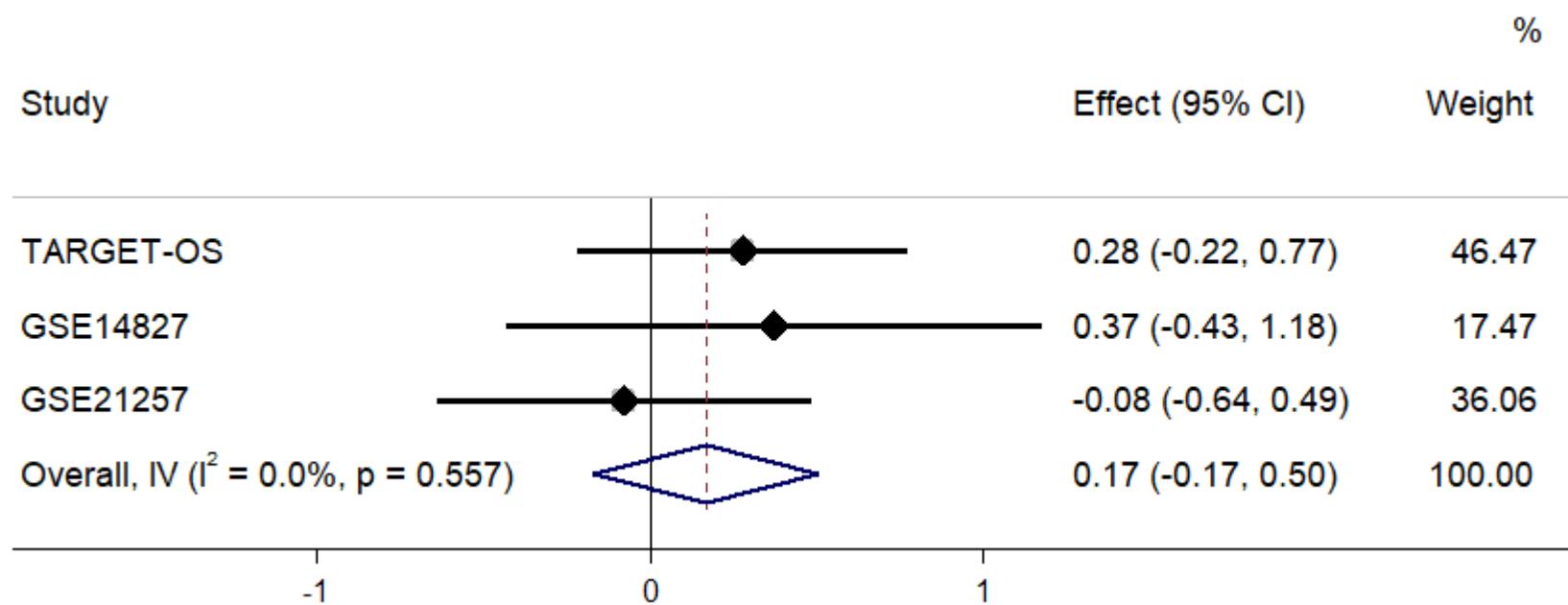

(b)

Supplement: Supplementary Materials — Figure S1: violin-plot displayed CKS1B expression in osteosarcoma (OS) and nontumor samples from in-house tissue microarrays and external microarrays. Figure S2: the ability of CKS1B expression of distinguishing OS from nontumor tissues in each microarray dataset. Figure S3: integrative analysis of CKS1B expression in osteosarcoma (OS) of each microarray dataset. Figure S4: the clinic-pathological significance of CKS1B in osteosarcoma (OS). Figure S5: integrated analysis of the prognostic value of CKS1B reflecting osteosarcoma (OS) patients' occurring metastasis and survival condition. Figure S6: Pearson's correlation analysis showing association between CKS1B and immune cells. Figure S7: Pearson's correlation analysis showing association between CKS1B and immune checkpoint genes. Figure S8: the expression matrix of 11 samples osteosarcoma (OS) patients was integrated by R package harmony and it showed that 11 samples have strong fitting degree after eliminating batch effect. Figure S9: scRNA-seq analysis process of GSE152048 before cell annotation. [file 7228584.f1.zip › 7228584.f1/Figure S5.pdf]

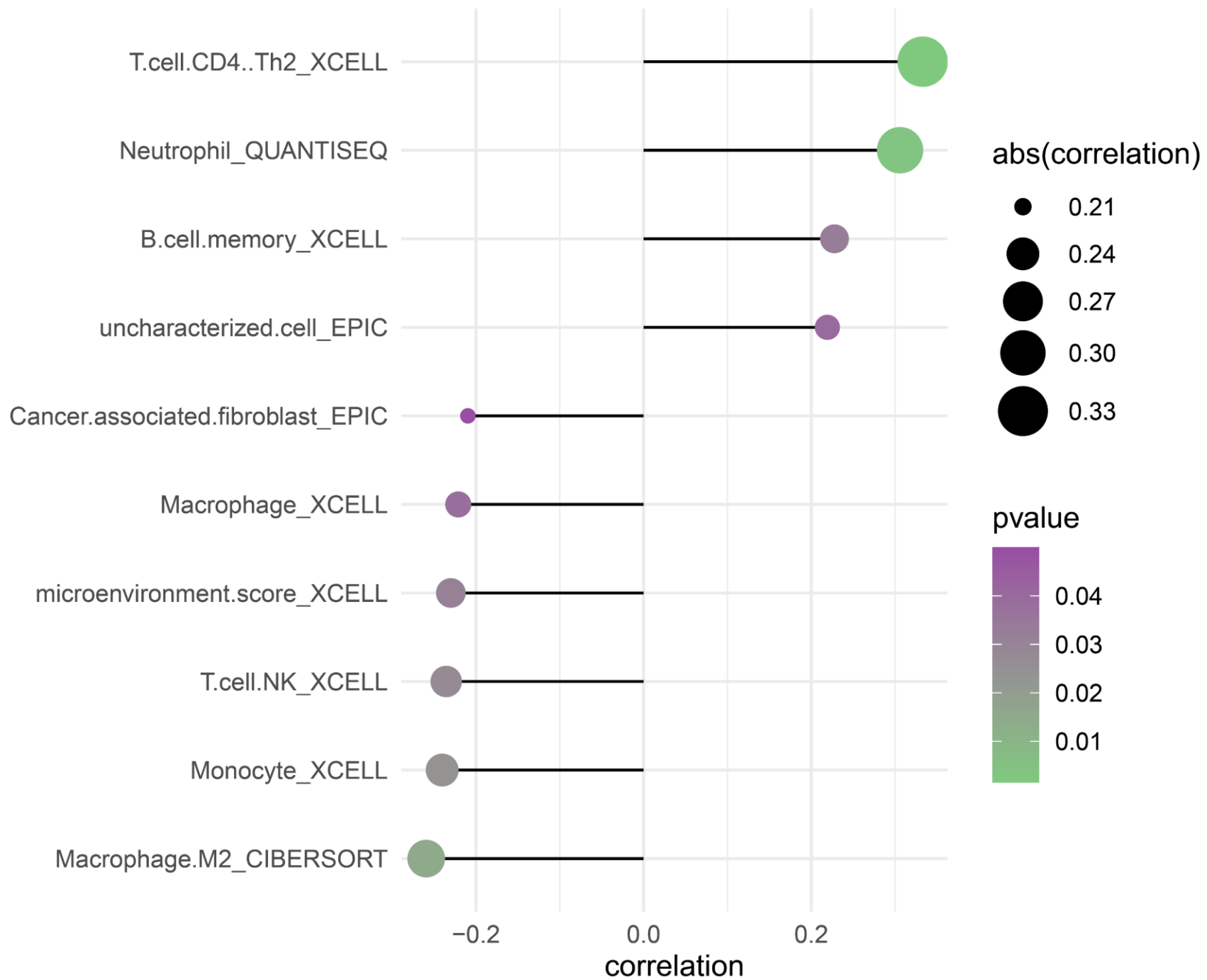

Supplement: Supplementary Materials — Figure S1: violin-plot displayed CKS1B expression in osteosarcoma (OS) and nontumor samples from in-house tissue microarrays and external microarrays. Figure S2: the ability of CKS1B expression of distinguishing OS from nontumor tissues in each microarray dataset. Figure S3: integrative analysis of CKS1B expression in osteosarcoma (OS) of each microarray dataset. Figure S4: the clinic-pathological significance of CKS1B in osteosarcoma (OS). Figure S5: integrated analysis of the prognostic value of CKS1B reflecting osteosarcoma (OS) patients' occurring metastasis and survival condition. Figure S6: Pearson's correlation analysis showing association between CKS1B and immune cells. Figure S7: Pearson's correlation analysis showing association between CKS1B and immune checkpoint genes. Figure S8: the expression matrix of 11 samples osteosarcoma (OS) patients was integrated by R package harmony and it showed that 11 samples have strong fitting degree after eliminating batch effect. Figure S9: scRNA-seq analysis process of GSE152048 before cell annotation. [file 7228584.f1.zip › 7228584.f1/Figure S6.pdf]

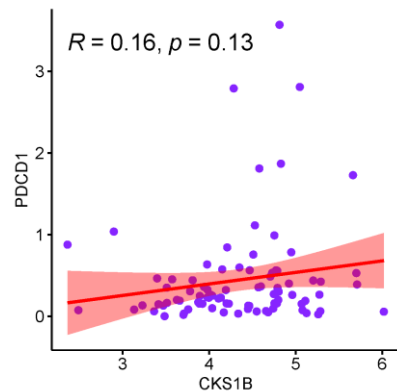

(a)

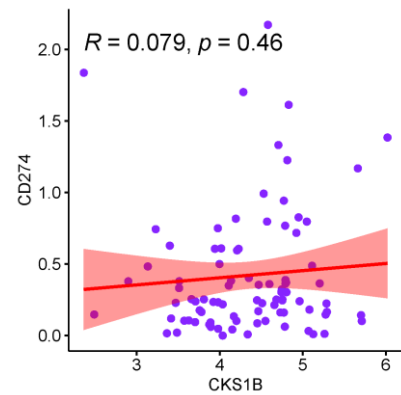

(b)

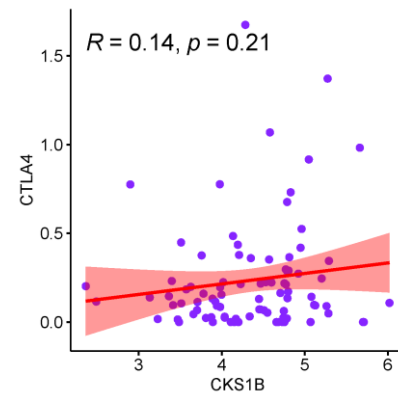

(c)

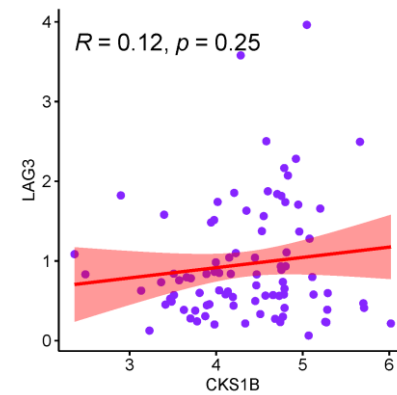

(d)

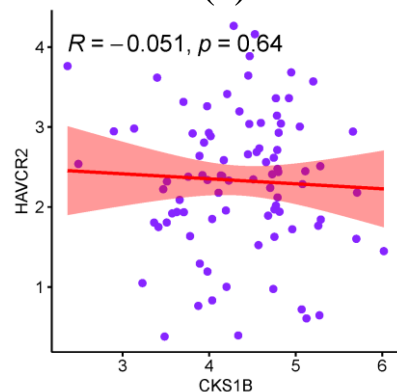

(e)

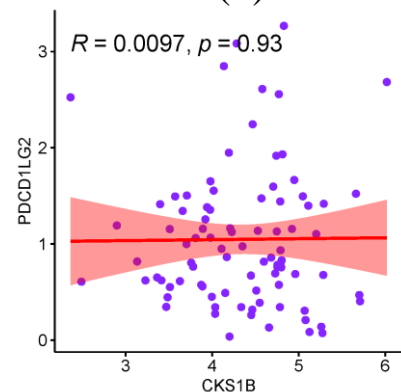

(f)

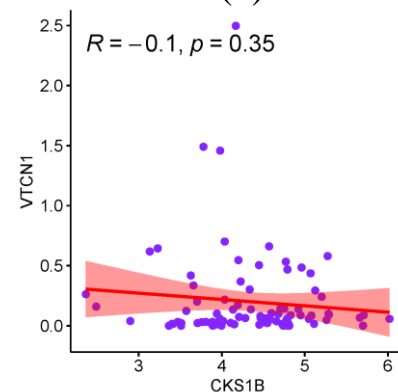

(g)

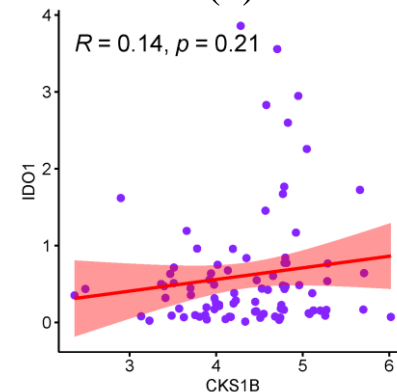

(h)

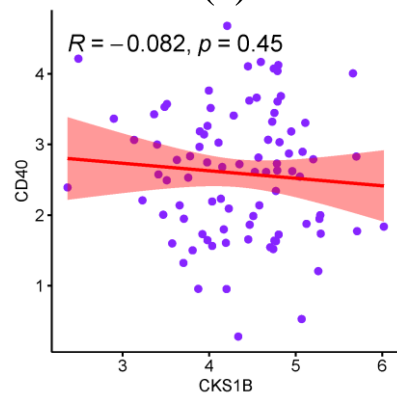

(i)

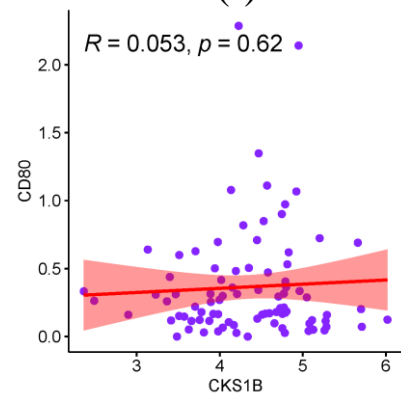

(j)

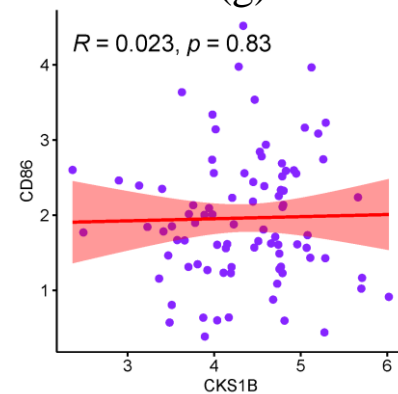

(k)

Supplement: Supplementary Materials — Figure S1: violin-plot displayed CKS1B expression in osteosarcoma (OS) and nontumor samples from in-house tissue microarrays and external microarrays. Figure S2: the ability of CKS1B expression of distinguishing OS from nontumor tissues in each microarray dataset. Figure S3: integrative analysis of CKS1B expression in osteosarcoma (OS) of each microarray dataset. Figure S4: the clinic-pathological significance of CKS1B in osteosarcoma (OS). Figure S5: integrated analysis of the prognostic value of CKS1B reflecting osteosarcoma (OS) patients' occurring metastasis and survival condition. Figure S6: Pearson's correlation analysis showing association between CKS1B and immune cells. Figure S7: Pearson's correlation analysis showing association between CKS1B and immune checkpoint genes. Figure S8: the expression matrix of 11 samples osteosarcoma (OS) patients was integrated by R package harmony and it showed that 11 samples have strong fitting degree after eliminating batch effect. Figure S9: scRNA-seq analysis process of GSE152048 before cell annotation. [file 7228584.f1.zip › 7228584.f1/Figure S7.pdf]

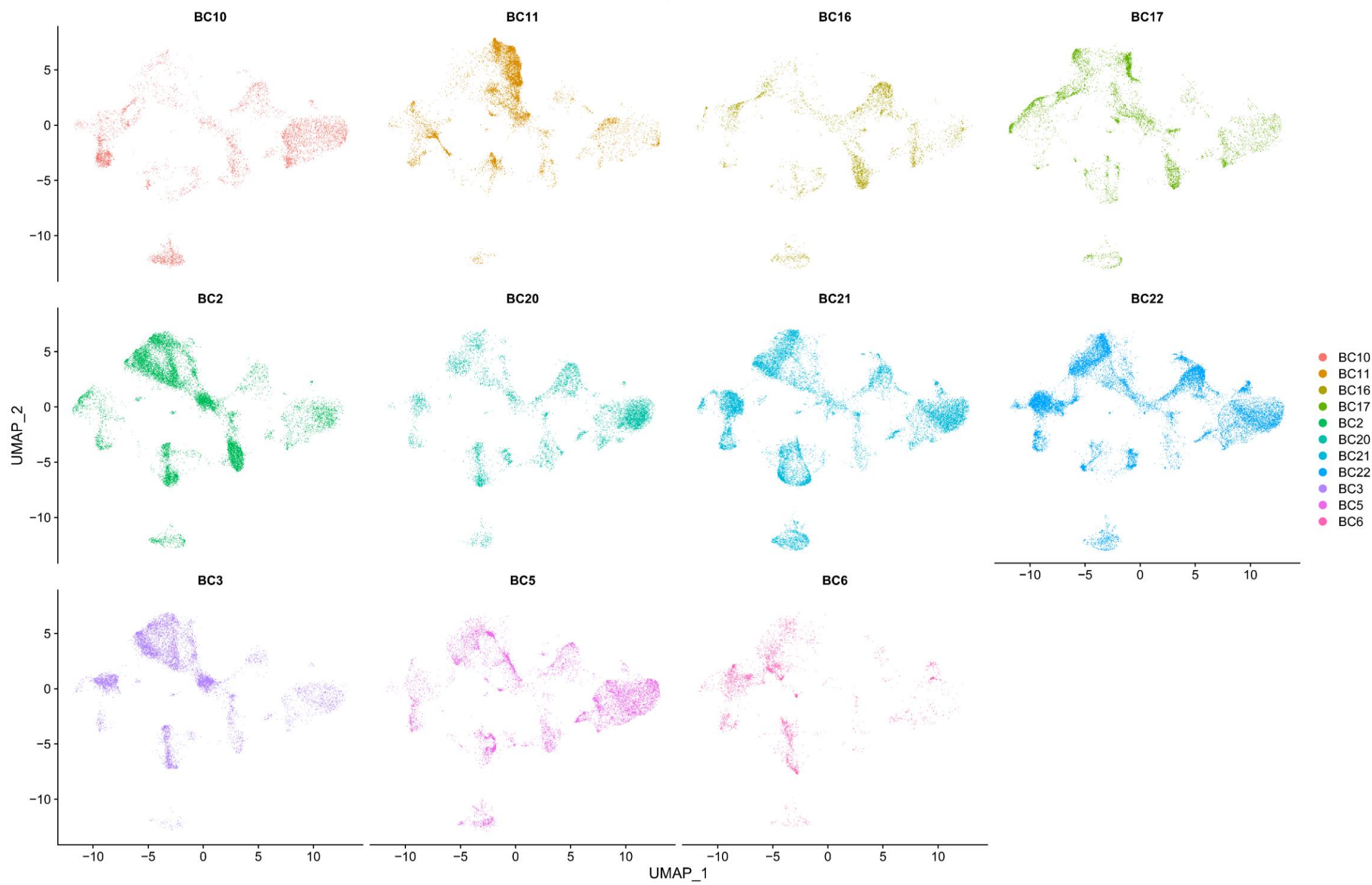

Supplement: Supplementary Materials — Figure S1: violin-plot displayed CKS1B expression in osteosarcoma (OS) and nontumor samples from in-house tissue microarrays and external microarrays. Figure S2: the ability of CKS1B expression of distinguishing OS from nontumor tissues in each microarray dataset. Figure S3: integrative analysis of CKS1B expression in osteosarcoma (OS) of each microarray dataset. Figure S4: the clinic-pathological significance of CKS1B in osteosarcoma (OS). Figure S5: integrated analysis of the prognostic value of CKS1B reflecting osteosarcoma (OS) patients' occurring metastasis and survival condition. Figure S6: Pearson's correlation analysis showing association between CKS1B and immune cells. Figure S7: Pearson's correlation analysis showing association between CKS1B and immune checkpoint genes. Figure S8: the expression matrix of 11 samples osteosarcoma (OS) patients was integrated by R package harmony and it showed that 11 samples have strong fitting degree after eliminating batch effect. Figure S9: scRNA-seq analysis process of GSE152048 before cell annotation. [file 7228584.f1.zip › 7228584.f1/Figure S8.pdf]

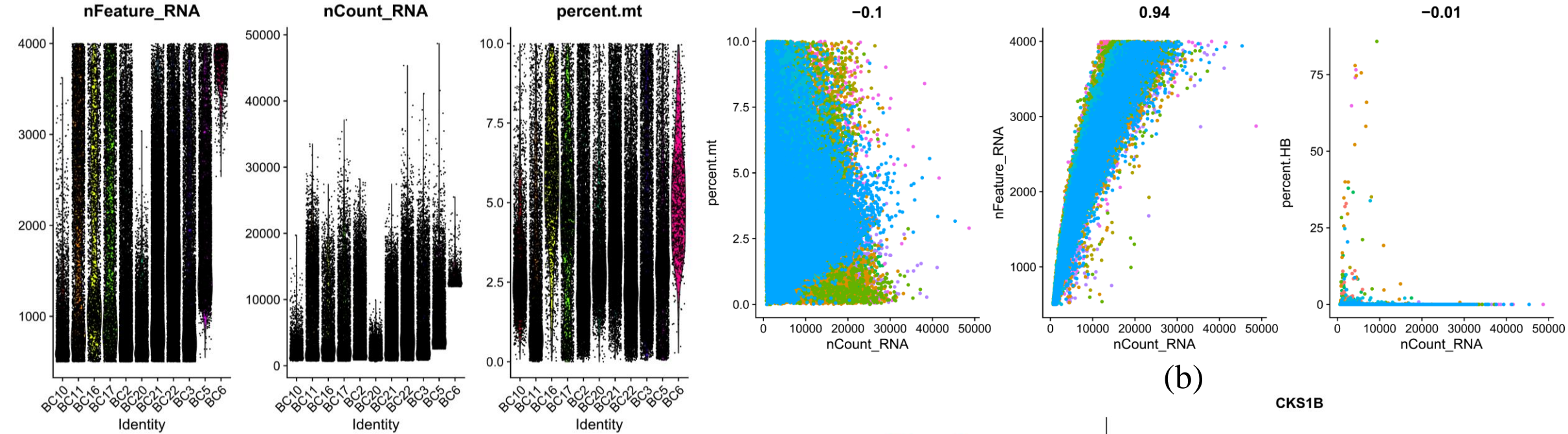

(b)

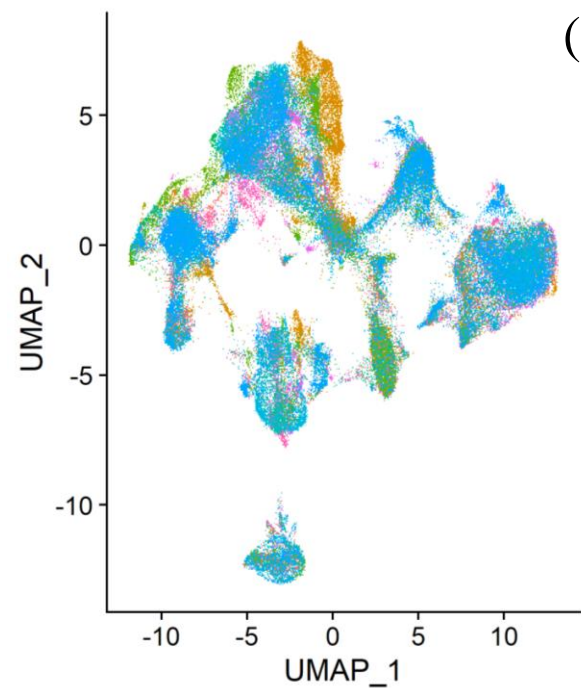

(a)

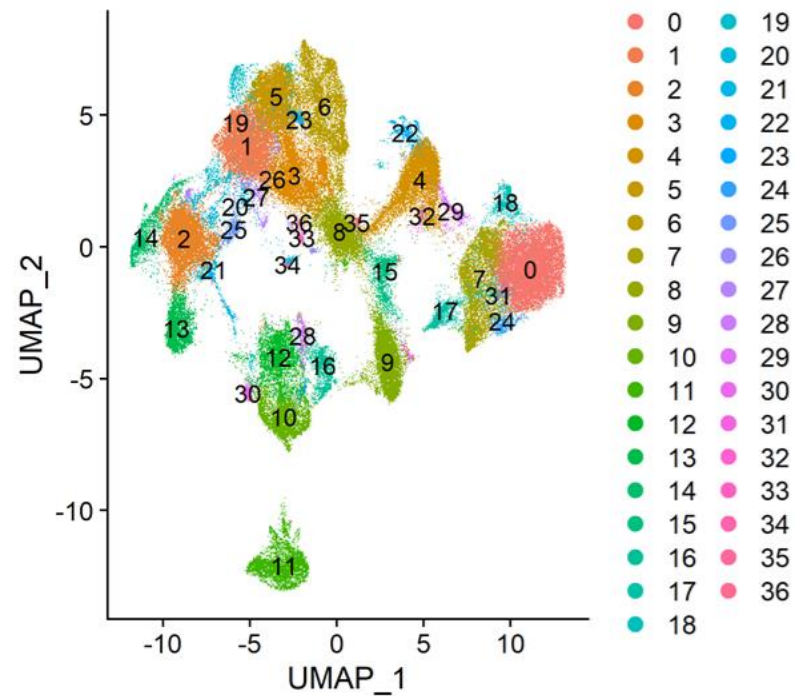

(d)

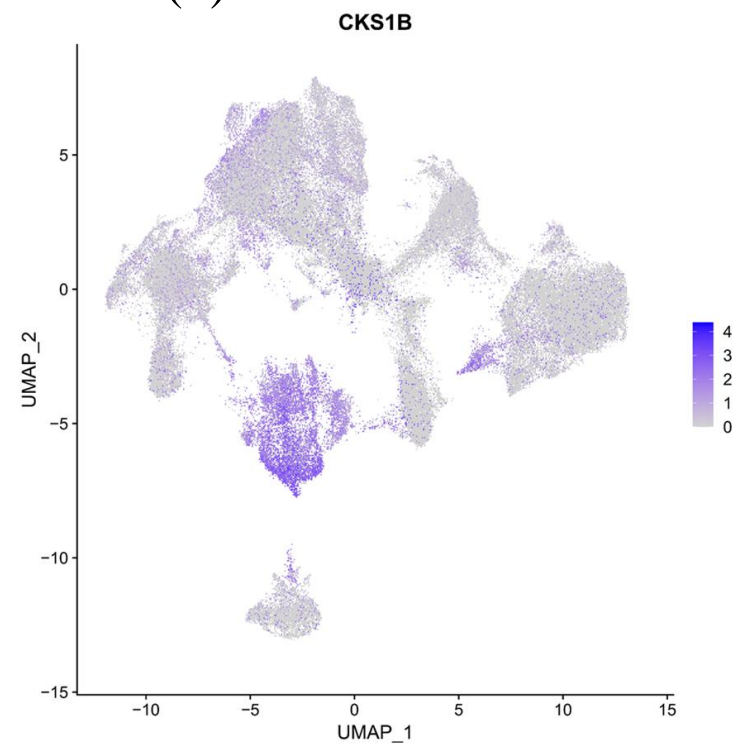

(e)

(c)

Supplement: Supplementary Materials — Figure S1: violin-plot displayed CKS1B expression in osteosarcoma (OS) and nontumor samples from in-house tissue microarrays and external microarrays. Figure S2: the ability of CKS1B expression of distinguishing OS from nontumor tissues in each microarray dataset. Figure S3: integrative analysis of CKS1B expression in osteosarcoma (OS) of each microarray dataset. Figure S4: the clinic-pathological significance of CKS1B in osteosarcoma (OS). Figure S5: integrated analysis of the prognostic value of CKS1B reflecting osteosarcoma (OS) patients' occurring metastasis and survival condition. Figure S6: Pearson's correlation analysis showing association between CKS1B and immune cells. Figure S7: Pearson's correlation analysis showing association between CKS1B and immune checkpoint genes. Figure S8: the expression matrix of 11 samples osteosarcoma (OS) patients was integrated by R package harmony and it showed that 11 samples have strong fitting degree after eliminating batch effect. Figure S9: scRNA-seq analysis process of GSE152048 before cell annotation. [file 7228584.f1.zip › 7228584.f1/Figure S9.pdf]
